# Effects of terrigenous organic substrates and additional phosphorus on bacterioplankton metabolism and exoenzyme stoichiometry

**DOI:** 10.1111/fwb.13593

**Published:** 2020-07-13

**Authors:** Tz‐Ching Yeh, Kathrin Krennmayr, Chien‐Sen Liao, Elisabet Ejarque, Jasmin Schomakers, Jr‐Chuan Huang, Franz Zehetner, Thomas Hein

**Affiliations:** ^1^ Institute of Hydrobiology and Aquatic Ecosystem Management University of Natural Resources and Life Sciences (BOKU) Vienna Austria; ^2^ WasserCluster Lunz (WCL) Biological Station Lunz am See Austria; ^3^ Department of Civil and Ecological Engineering I‐Shou University Kaohsiung Taiwan; ^4^ Institute of Soil Research University of Natural Resources and Life Sciences (BOKU) Vienna Austria; ^5^ Department of Geography National Taiwan University (NTU) Taipei Taiwan

**Keywords:** aquatic metabolism, bamboo forest soil, evergreen forest soil, laboratory experiment, terrigenous organic matter

## Abstract

1. Bamboo, as a pioneer vegetation, often forms forests on bare lands after catastrophic landslides. Compared to evergreen forest soil, bamboo forest soil is much more labile, with a higher percentage of microbially derived organic carbon (OC), lower molecular weight, and lower humic acid content. We hypothesised that different terrigenous organic matter (tOM) sources with varying lability and phosphorus (P) availability select for bacterioplankton with distinct metabolic pathways.
2. We incubated natural bacterioplankton assemblages with tOM leached from bamboo forest soil (BOM) and evergreen forest soil (EOM) and compared these to a lake water control. To test if microbial metabolism would be limited by OC or P availability of each tOM treatment, we used acetate as an extra labile OC source and phosphate as an inorganic P source. Bacterial metabolism was measured by analysing respiration via O_2_ consumption and production via tritiated thymidine (TdR) assimilation.
3. Bacterioplankton metabolism is limited by the availability of P in BOM substrates. When using BOM, bacteria had higher enzymatic activities for phosphatase. The nutrients required for bacterial biomass seemed to be derived from organic matter. Under BOM treatment, bacterial production (BP) (0.92 ± 0.13 μg C L^−1^ hr^−1^) and cell specific TdR assimilation rates (0.015 ± 0.002 10^–18^ M TdR cell^−1^ hr^−1^) were low. Adding P enhanced BP (BOM_+P_ 1.52 ± 0.31 and BOM_+C+P_ 2.25 ± 0.37 μg C L^−1^ hr^−1^) while acetate addition had no significant effect on BOM treatment.
4. This indicated that the bacteria switched to using added inorganic P to respire a P‐limited BOM substrate, which increased total BP and abundance, resulting in even more active respiration and lower growth efficiency. We also found higher activities for chitin‐degrading enzyme β‐N‐acetylglucosaminidase, which is associated with N mining from aminosaccharides.
5. Microbes using EOM, however, did not change metabolic strategies with additional acetate or/and inorganic P. This is due to higher concentrations of organic P in EOM substrates and the presence of inorganic N in the EOM leachates an alternative nutrient source. Bacteria produced β‐glucosidase and leucyl‐aminopeptidase in order to utilise the humic substances, which sustained greater bacterial abundance, higher BP (2.64 ± 0.39 μg C L^−1^ hr^−1^), and lower cell‐specific respiration. This yielded a much higher bacterial growth efficiency (15 ± 9.2%) than the lake water control.
6. Our study demonstrated the aquatic metabolic discrepancy between tOM of different forest types. Bacterioplankton in BOM and EOM exhibit distinct metabolic responses. Bacterial metabolic strategy when using BOM implied that the supposedly stabilised biomass OM might be efficiently used by aquatic bacterioplankton. As the labile and nutrient‐deficient BOM is more susceptible to the influence of additional nutrients, fertiliser residues in bamboo forest catchments might have a stronger effect on aquatic bacterial metabolic pathways. Thus, it is important to take tOM differences into consideration when building models to estimate soil carbon turnover rates along a terrestrial–aquatic continuum.

## 1 INTRODUCTION

An estimated 1.9 Pg soil‐derived organic carbon (OC) is released from land to inland waters each year (Regnier et al., 2013). This complex terrigenous organic matter (tOM) accounts for 89% of the bulk dissolved OC in freshwater systems (Guillemette, McCallister, & del Giorgio, 2016). Terrigenous organic matter and primary production both influence aquatic ecosystem processes by providing varying degrees of carbon, nutrients, and energy (Tranvik, 1988), yet we are just starting to understand the importance of tOM quality for aquatic microbial metabolism. Heterotrophic bacterioplankton is a major player in the regulation of biogeochemical cycles of carbon and nutrients in lentic ecosystems. Lake bacteria can respire tOM (Karlsson, Jansson, & Jonsson, 2007; McCallister & del Giorgio, 2008), repackaging a portion of it into bacterial biomass (Kritzberg, Cole, Pace, Granéli, & Bade, 2004) and further transferring it into aquatic food webs (Berggren, Ström, et al., 2010; Guillemette et al., 2016). Different forest types can provide aquatic ecosystems with tOM of varying quality, some with more labile carbon sources or higher nutrient values than others, thus potentially impacting aquatic bacterial metabolism. However, past studies found that tOM from different woody forests had no effect on carbon‐specific respiration due to a slight decoupling of catabolic and anabolic respiratory reactions or the inherent variability associated with respiration measurements (e.g. Lennon & Pfaff, 2005).

Our study compares the effects of tOM from woody and bamboo forests on the metabolism of aquatic bacteria. Belonging to the grass family, bamboo is an important forest ecosystem and natural resource in Asia, Africa, and South America. After an initial lag phase, bamboo forests can effectively accumulate around 2 Mg C ha^−1^ year^−1^ soil OC on bare lands after catastrophic landslides, whereas it would take several decades for evergreen woody forests to re‐establish effective populations for soil OC sequestration (Schomakers et al., 2017). The chemical composition of soils differs between bamboo and woody forests: bamboo soils are characterised by a lower molecular weight and lower aromatic content OC pool compared to the high molecular weight (HMW) and high aromatic content OC of evergreen forest soils (Wang, Tian, & Chiu, 2016). Moreover, the ratio of microbially derived OC to ambient soil OC is especially high in bamboo forest soils compared to that of evergreen woody forests (Chang & Chiu, 2015), indicating that the former possesses higher levels of labile OC (Sparling, 1992). Although phosphatase activity appears to be similar between the two forest soil types (Chang & Chiu, 2015), previous studies have shown that bamboo soils have a lower level of total phosphorus (P) than woody forest soils (J. Schomakers, personal communication). Additionally, inorganic phosphorus from the forest floor of mountainous catchments provides aquatic systems with an important P flux that may increase with enhanced frequency and intensity of typhoons (Lee, Huang, Kao, & Tung, 2013), potentially influencing aquatic bacterial metabolism.

Determining how microbes allocate OC—i.e. how they balance molecule breakdown (e.g. for energy production) and build‐up (e.g. for biomass production)—is particularly important for the understanding of organic matter (OM) turnover and fate (Schimel & Schaeffer, 2012). Bacteria have different strategies of resource use to cope with heterogeneous substrates, for instance, the selective use of labile OC (e.g. algal‐derived source) for respiration and recalcitrant OC (e.g. lignin‐derived source) for biomass production (Guillemette et al., 2016). Which pathways are selected depends on the chemical properties and accessibility of the consumed substrate in the environment (Russell, 2007) as well as the energy and stoichiometric requirements of cells (Vallino, Hopkinson, & Hobbie, 1996). Nutrient limitation and the trophic basis of bacterioplankton production can be understood by focusing on exoenzyme activities and their relationships with OM substrate availabilities or microbial production dynamics (Foreman, Franchini, & Sinsabaugh, 1998).

Complex tOM polymers often stimulate exoenzyme activities that catalyse the degradation of tOM into dimers and monomers, simple units that are then used in metabolic pathways (Chróst, 1990). The processes that determine metabolic rates are constrained by the chemical properties of the available organic substrates, such as humic content (Moran & Hodson, 1990), mean molecular weight (Weiss & Simon, 1999), the stoichiometry of growth‐limiting nutrients (Hunt, Parry, & Hamilton‐Taylor, 2000), and oxidation state (Vallino et al., 1996). To maintain biomass stoichiometry, microbial communities may adapt their foraging strategies to the available substrates (Sinsabaugh, Manzoni, Moorhead, & Richter, 2013). For instance, β‐glucosidase (BG) activity would increase in response to cellobiose presence and decrease in response to glucose presence, and leucyl‐aminopeptidase activity would increase in response to available proteins (Foreman et al., 1998). When there are low degrees of dissolved inorganic nitrogen and phosphorus availability, carbon acquisition is constrained by exoenzymes that can mine N and P from organic substrates (Foreman et al., 1998). Therefore, by comparing bacterial production (BP)‐normalised N and P exoenzyme activities in relation to that of C (e.g. Clinton, Edwards, & Findlay, 2010; Sinsabaugh, Van Horn, Shah, & Findlay, 2010), we can investigate the metabolic constraints of nutrient acquisition in different tOM substrates.

In our 10‐day laboratory incubation study, we compared the bacterial metabolic and enzymatic activities associated with C, N, and P acquisition using soil leachate from either bamboo (BOM) or evergreen woody forest (EOM) as the main tOM substrate. To test the degree of OC lability and P availability, and which enzymes were generated by bacteria to acquire nutrients in each tOM substrate, we further added acetate as an extra labile OC and phosphate as an inorganic P source to each tOM. We hypothesised that (1) bacterioplankton use different metabolic pathways in BOM or EOM substrates due to differences in OC quality and their biological availability. We compared the metabolic activities in lake water control, BOM, and EOM substrates before and after adding acetate, a simple C source. It is a common lower molecular weight compound in aquatic systems (Johnson & Tank, 2009) in addition to tOM‐derived OC. Berggren, Laudon, Haei, Ström, and Jansson (2010) showed that acetate exported from forests is a main component of the aquatic labile OC pool, constituting 45% of total bacterial consumption. Although labile OC is typically incorporated into biomass with low efficiencies (Linton & Stephenson, 1978), it potentially provides a significant carbon and energy source for bacteria (del Giorgio & Cole, 1998). In addition to substrate quality, the availability of inorganic nutrients is another key factor limiting bacterial growth (del Giorgio & Cole, 1998). Thus, we also hypothesised that (2) bacterioplankton using BOM substrate will have increased phosphatase activities compared to EOM in order to meet metabolic requirements. Lennon and Pfaff (2005) found that dissolved organic phosphorus is a major driver of bacterioplankton productivity in different types of temperate deciduous–coniferous forest soil leachates. To investigate how bacteria use P in BOM treatments, we compared their metabolic activities in lake (control), BOM, and EOM substrates with and without additional inorganic phosphorus.

## 2 METHODS

### 2.1 Field sampling

Two batches of 10‐day microcosm experiments were conducted using filtered lake water (LAKE) with bamboo forest A‐horizon soil‐derived OM (BOM, 26 October–5 November 2015) and evergreen forest soil (EOM, 9–19 November 2015). Surface water (depth *c*. 0.5 m) of the oligotrophic lake Lunz (Austria), was used after filtration (GF/C Whatman, 1 μm pore size, VWR international GmbH, pre‐combusted at 450°C, 4 hr) to reduce particles and grazing pressure (Moran & Hodson, 1990). The collected water was acclimated inside a climate chamber (20°C) for 24 hr prior to the 10‐day experiments.

The topsoil layer (0–10 cm) was collected from a bamboo forest that had established on a 1989 landslide scar, and from a reference evergreen woody forest in the Tsengwen reservoir catchment in Alishan Mountain Range, Taiwan (Schomakers et al., 2017). Currently, this area is low in agricultural activity (6.9% of the total area) but very landslide prone; 3% of the catchment area was bare‐lands resulting from previous landslide events (J. C. Huang, personal communication, 2018). Soils were air‐dried, gently ground, and homogenised with mortar and pestle to disintegrate the soil aggregates and sieved to 2 mm. To simulate the process of leaching during erosion and transport during landslide events, each type of soil (30 g) was soaked in 1 L autoclaved Milli‐Q water and stirred for 48 hr (20°C, 650 rpm) to induce the leaching phase. The slurry was subsequently centrifuged and filtered (GF/F Whatman, 0.7 μm pore size, pre‐combusted at 450°C, 4 hr); the leachates were freeze‐dried (Freeze drier BenchTop6K v. Labor partner GmbH; Sun, Perdue, Meyer, & Weis, 1997) and stored at −80°C before use. Data showed no significant changes in the optical properties before and after freeze‐drying of the soil leachates (Table S1).

### 2.2 Experimental setup

Cylinder‐shaped acrylic vessels (650 ml, Seitzberger GmbH) were used in the experiments and kept on a horizontal shaker (100 rpm) to guarantee well‐mixed conditions and prevent the formation of biofilms and anoxic conditions. The incubation was conducted in the dark inside a 20°C‐climate chamber (SimTech SKZ 020‐s) to minimise the effect of primary production and photo‐degradation of dissolved OM. Approximately 1.5 mg freeze‐dried BOM or EOM substrate was added and dissolved well into each incubation vessel filled with lake water; similar dissolved OC (DOC) concentrations were yielded on Day 0 in the two tOM treatments, which were significantly higher than that of the LAKE (Figure S1). LAKE‐only control treatments (LAKE_control_) from the two batch experiments were pooled for data analysis, because the water used in the two batch experiments was not significantly different in terms of physicochemical properties and microbiological activities (Table S2). The amendment of labile OC and P was conducted using acetate and phosphate, respectively, and the concentrations used were derived from previous studies focusing on stimulating microbial metabolic activities (e.g. Berggren, Laudon, et al., 2010; Ghosh & Leff, 2013; Guenet et al., 2014; Steen, Quigley, & Buchan, 2016) with some modifications according to previous typhoon field observations (Yeh et al., 2018). Table 1 shows the complete experimental setup with 3 OM substrates (LAKE, BOM, and EOM) and their four nutrient additions (control, +C, +P, and +C+P), which was designed to test the respective first and second hypotheses. Each of the batch experiments had a LAKE_control_. Nutrient addition to LAKE was conducted along with the EOM batch. In total, there were 13 different OM substrate × nutrient addition combinations, and each had three experimental replicates, resulting in *n* = 39. The +C treatments received 120 µl sodium acetate (C_2_H_3_NaO_2_, 0.1 M, Sigma Aldrich) and the +P treatments received 1,300 µl sodium dihydrogen phosphate (NaH_2_PO_4_, 1 mM, Sigma Aldrich), resulting in 18.4 μM C and 2 μM P final concentration in the experiment vessels, respectively. The +C+P treatment received both the respective C and P amounts.

**TABLE 1:** Experimental setup

Treatment	Organic matter substrate		
	LAKE	BOM	EOM
Control	LAKE_control_	BOM_control_	EOM_control_
+C	LAKE_+C_	BOM_+C_	EOM_+C_
+P	LAKE_+P_	BOM_+P_	EOM_+P_
+C + P	LAKE_+C+P_	BOM_+C+P_	EOM_+C+P_

Abbreviations: +C, acetate addition; +P, phosphate addition; BOM & EOM, dissolved organic matter derived from bamboo‐ and evergreen forest soil; LAKE, lake water.

### 2.3 Laboratory analyses

Samples were analysed on Day 0, 1, 2, 3, 4, 7, and 10, except for respiration which was measured only on Day 0, 4, 7, and 10. Water was filtered (GF/F Whatman, 0.7 μm pore size, VWR international GmbH, pre‐combusted at 450°C, 4 hr) for solute analysis. Concentrations of ammonium, nitrite, nitrate, and phosphate were analysed on a continuous flow analyser FLOWSYS RA104 (Alliance Instr.) with a detection limit of 4, 1, 20, and 2 µg/L, respectively. Dissolved OC was measured on a portable TOC analyser (Sievers 900, GE) with a detection limit of 0.2 mg/L.

Each sample was scanned for absorbance at 200–800 nm on a spectrophotometer (Hitachi U‐2900) and for fluorescence properties on a fluorospectrometer (Hitachi F‐7000) using a 1‐cm quartz cuvette. Excitation–emission matrix (EEM) were generated via scanning the samples at excitation (Ex) wavelengths between 200–450 nm at 5 nm steps and emission (Em) wavelengths between 250–600 nm at 2 nm steps, at a scanning speed of 12,000 nm/min. Wavelength‐dependent inefficiencies of the detection system where corrected using the manufacturer’s built‐in correction methods. Deionised water EEMs were measured each day of analysis in triplicate, and the average subtracted to the sample EEM as a blank. Also, the area under the Raman peak (Em = 371–428 nm, Ex = 350 nm) was used to transform raw fluorescence data into Raman units (Lawaetz & Stedmon, 2009). Absorbance measurements were used to correct for inner‐filter effects according to Lakowicz (2006). Parallel factor analysis was used to model the changes in optical properties of dissolved OM using the drEEM toolbox for MATLAB (Murphy, Stedmon, Graeber, & Bro, 2013) (The MathWorks, Inc.). Preliminary models were fit for 2–9 components, and the best model was chosen according to the following criteria: (1) modelled EEMs with pattern‐less residuals; (2) chemically meaningful spectral loadings; and (3) split‐half validation using for data splits, six combinations, and three validation tests (S4C6T3). Parallel factor analysis components were presented as percentages of the total fluorescence. Moreover, two indices were used, i.e. biological index (BIX, Em intensity at 380 nm divided by the maximum Em intensity between 420 and 435 nm, at Ex 310 nm, Huguet et al., 2009) which is positively correlated to freshly released dissolved OM, and the absorbance ratio E_2_: E_3_ index (absorbance measured at wavelengths 250 nm divided by that at 365 nm), which is inversely related to aromaticity and average molecular weight (Peuravuori & Pihlaja, 1997).

Non‐filtered water samples were analysed for bacterial abundance (BA), bacterial respiration (BR), BP, and enzyme activity. For abundance analysis, samples were formaldehyde‐fixed (2% final concentration, Sieczko & Peduzzi, 2014), shock‐frozen in liquid nitrogen and stored at −80°C prior to further analysis. Stained microbial cells (SYTOX^©^ Dead Cell Stains, Invitrogen, 2.5 μM final concentration) were counted by a flow cytometer (CytoFLEX, Beckman Coulter) and validated by direct count on an epifluorescence microscope (LSM 710, Zeiss); in the latter, 20 ocular fields and a minimum of 200 cells were counted for each sample.

The consumption rate of dissolved oxygen on OM substrate degradation was measured as a surrogate for BR rate following the method of Warkentin, Freese, Karsten, and Schumann (2007). Sub‐samples from each experiment vessel were measured inside a clean 100‐ml Schott bottle (Duran Group) equipped with a planar optode (Presens), and were incubated in the same conditions as the experiment vessel. The dissolved oxygen concentration was measured at 0, 30, 60, and 90 min. Respiration rate (mg O_2_ L^−1^ hr^−1^) was calculated as the slope of dissolved oxygen decrease over the 90‐min incubation time, and the rate was converted to C units (μg C L^−1^ hr^−1^) assuming a respiratory quotient of 1 (del Giorgio & Cole, 1998; Smith & Prairie, 2004).

Bacterial production was measured by the incorporation rate of ^3^H‐thymidine (^3^H‐TdR) into the DNA of heterotrophic bacterioplankton cells, following the microcentrifugation method (Kirchman, 2001). Methyl‐^3^H‐thymidine with a specific activity of 80 Ci/mmol (Amersham Biosciences) was used as the radioactive tracer (20 nM final concentration) which showed a constant isotope dilution level (Pollard & Moriarty, 1984). Two analytic replicates and one sterilised control (100% trichloroacetic acid) were assayed for each sample (20°C, in the dark) and photon emission was counted on a scintillation counter (Beckman Coulter LS6500). Specific conversion factors for heterotrophic bacterioplankton were used: 2.4 × 10^18^ μg of C/mol thymidine for carbon conversion constant (Fuhrman & Azam, 1982); 20 fg for carbon content per bacterial cell (Cho & Azam, 1988). We used common conversion factors; however, there might be substantial uncertainty in these factors since we did not directly measure the thymidine–carbon conversion rate nor the mean carbon content of a cell. Bacterial growth efficiency was calculated using the formula: bacterial growth efficiency = BP/(BP + BR) and presented as a percentage.

Samples were fluorometrically assayed for five hydrolase activities following the method described in Sieczko and Peduzzi (2014) and measured on a 96‐well microplate reader (Varioskan Flash, Thermo Fisher Scientific). Table 2 presents the five hydrolases assayed in this study and the chemical reaction they catalyse (substrate concentrations range 250–500 μM). Standards 4‐methylumbelliferone (M1381, Aldrich) and 7‐amino‐4‐methylcoumarin (257370, Aldrich) were mixed with samples using various concentrations (0, 0.1, 0.5, 1, 2, 5 µM). After incubation (1 hr, 20°C, in the dark), enzyme activity within the linear increasing phase was calculated as the difference between values read at T_1_ and T_0_ and expressed in nM/hr. Metabolic effort (MEF) of each enzyme was calculated by dividing enzymatic activity by the BP (Sieczko & Peduzzi, 2014). Normalising enzyme activity allows us to directly compare the scaling relationships of enzyme activities (Sinsabaugh et al., 2010) across treatment combinations.

**TABLE 2:** Hydrolysis enzymes used in this study and the related catalytic reactions

Enzyme name	EC number^a^	Substrate	Catalysis action	Reference
β‐glucosidase (BG)	EC 3.2.1.21	4‐MUF‐β‐D‐glucopyranoside	Hydrolysis of the β‐glycosidic bonds; releases glucose	Mondini, Cayuela, Sanchez‐Monedero, Roig, and Brookes (2006)
Cellobiohydrolase (CBH)	EC 3.2.1.91	4‐MUF‐β‐D‐cellobioside	Hydrolysis of the non‐reducing ends of the cellulose chain; releases cellobiose	Carreiro, Sinsabaugh, Repert, and Parkhurst (2000)
Alkaline phosphatase (AP)	EC 3.1.3.1	4‐MUF‐phosphate	Hydrolysis phospholipids and phosphosaccharides; releases phosphate ion	Mondini et al. (2006)
β‐*n*‐acetylglucosaminidase (NAG)^b^	EC 3.2.1.52	4‐MUF‐*N*‐acetyl‐β‐D‐glucosaminide	Hydrolysis of aminosaccharides from chitin or similar molecules	Parham and Deng (2000)
Leucyl‐aminopeptidase (LAP)	EC 3.4.11.1	L‐Leucine‐AMC hydrochloride	Hydrolysis of peptide bonds adjacent to leucine and other amino acids	Carreiro et al. (2000)

^a^ Enzyme commission number.

^b^ The entry of N‐acetyl‐b‐D‐glucosaminidase was also listed as β‐N‐acetylhexosaminidase.

### 2.4 Statistical methods

Repeated measures analysis of variance (rmANOVA) was performed in the software IBM SPSS (v.21, IBM corp.) to compare means of variables that are based on repeated observations. Bacterial metabolic parameters were dependent variables (DVs); sampling time (Day 0, 1, 2, 3, 4, 7, and 10) was a within‐subjects factor; and OM substrate (LAKE, BOM, EOM) and nutrient addition (control, +C, +P, +C+P) were between‐subject factors. The statistical design aimed to answer our research questions; i.e. (1) if the mean of BOM_control_ or EOM_control_ was significantly different from that of the LAKE_control_; and (2) within each OM substrate, if the mean of the +C, +P, or +C+P treatment was significantly different from that of the control at different time points of the experiment. Levene’s test was used for testing if the error variance of the DV was equal across groups. If the assumption of sphericity was violated (Mauchly’s test), the degrees of freedom were corrected using Greenhouse–Geisser estimates of sphericity. Post‐hoc tests were conducted using Bonferroni confidential interval adjustment. Main effects of the between‐subjects variables (OM substrate or/and nutrient addition) were reported if there was no interaction between OM substrate and nutrient addition on the DV. Otherwise, the dataset was split according to between‐subject variables and tested for simple main effect. Standardised major axis (SMA) and redundancy analysis (RDA) were analysed in R (version 3.5.2) and the software RSTUDIO (version 1.1.383, RStudio, Inc.). Standardised major axis was conducted in the R package *smatr* (Warton, Duursma, Falster, & Taskinen, 2012); this allowed us to describe the line‐of‐best‐fit that summarised the relationship between two variables (Warton et al., 2012). We used *smatr* and ANCOVA of log‐transformed enzyme MEF data to compare the scaling of MEF_AP_, MEF_NAG_, and MEF_LAP_ in relation to MEF_BG+CBH_. The disparity of C:N versus C:P nutrient acquisition slopes was analysed using pooled enzyme data (C‐hydrolase = MEF_BG+CBH_ and N‐hydrolase = MEF_NAG+LAP_) as described in Sinsabaugh et al. (2010); as well as the scaling between metabolic responses and OM properties. The residual plots of the SMA regression can be found in Figure S2. Redundancy analysis was used to test the prediction ability of explanatory variables (OM substrate and nutrient addition) on response variables (metabolism) and was conducted in the *vegan* package (Oksanen et al., 2019). Automatic stepwise model building was used to find out the most important explanatory variables in the RDA model depending on the values of Akaike information criterion; insignificant contribution was excluded. The model was tested using the ANOVA‐like permutation test *anova.cca* (Oksanen et al., 2019).

## 3 RESULTS

### 3.1 Terrigenous OM characterisation

Bamboo forest and evergreen forest A‐horizon soil had similar pH (Table 3). However, evergreen forest soil had higher electrical conductivity, and higher percentage of soil OC and total N. The amount of total P (organic and inorganic) in the evergreen forest soil was almost twice as high as those of the bamboo forest soil.

**TABLE 3:** Physicochemical properties of the soils used in experiment

Soil type	pH (H20)	Electrical conductivity (µS/cm)	Organic carbon (%)	Total nitrogen (%)	Total phosphorus (µg/g)	Organic phosphorus (µg/g)	Inorganic phosphorus (µg/g)
Bamboo forest A‐horizon soil (BOM)	4.9	108	5	0.3	446	358.9	87.1
Evergreen forest A‐horizon soil (EOM)	4.2	246	9.41	0.74	836.8	686	150.8

### 3.2 Quantity and quality of carbon and nutrient solutes

Dissolved OC concentrations of BOM_control_ and EOM_control_ were similar at Day 0 (Figure S1); both of their means were higher than LAKE_control_ (rmANOVA, *p* < 0.001). Nutrient addition had no effect on DOC concentration of any OM source. Nitrate‐N concentration had similar means across different OM sources and nutrient additions. However, EOM had significantly higher ammonium‐N concentration than BOM or LAKE regardless of nutrient addition (Table 4). Treatments + P and +C+P showed the greatest changes in phosphate concentrations in each OM substrate. However, EOM also had higher phosphate‐P concentration than LAKE on Day 0, while BOM and LAKE had similar values (Figure S1).

**TABLE 4:** Descriptive statistics of dissolved organic carbon (DOC), ammonium‐N, nitrate‐N, phosphate‐P concentrations; humic‐, fulvic acid‐, and protein‐like dissolved organic matter property as a percentage of the total fluorescence intensity; biological index (BIX), and E_2_: E_3_ molecular weight index. Means ± 1 *SEM*, *n* = 21. Bold and italic letters and asterisks denoted the statistical differences (*p* < 0.05, repeated measures ANOVA) among OM substrates and OC or P addition, respectively

OM	Addition	DOC (mg/L)	Ammonium‐N (μg/L)	Nitrate‐N (μg/L)	Phosphate‐P (μg/L)	Humic‐like (%)	Fulvic‐like (%)	Protein‐like (%)	BIX	E_2_: E_3_
LAKE	Control	2 ± 0.09 ***b***	21.6 ± 1.93 ***b***	300.3 ± 27.99 ***a***	0.6 ± 0.11 ***a***	36.09 ± 0.39 ***c***	33.8 ± 0.52 ***b***	30.11 ± 0.82 ***a***	0.84 ± 0.01 ***a***	6.44 ± 0.16 ***a***
	+C	2.04 ± 0.06	26.24 ± 2.55	331 ± 32.25	0.68 ± 0.26	37.87 ± 0.7	34.62 ± 0.72	27.5 ± 0.51	0.81 ± 0.01	6.28 ± 0.43
	+P	1.83 ± 0.09	21.64 ± 4.91	296.8 ± 30.43	26.74 ± 4.63*	37.4 ± 0.39	35.51 ± 0.5	27.09 ± 0.71	0.81 ± 0.01	5.45 ± 0.19
	+C+P	1.77 ± 0.06	27.02 ± 3.74	307.9 ± 31.22	23.22 ± 2.25*	37.56 ± 0.3	36.17 ± 0.35	26.27 ± 0.45	0.81 ± 0.01	6.21 ± 0.29
BOM	control	3.36 ± 0.29 ***a***	29 ± 5.58 ***b***	339.7 ± 52.48 ***a***	0.85 ± 0.2 ***a***	38.8 ± 0.36 ***b***	33.83 ± 0.3 ***c***	27.37 ± 0.5 ***a***	0.75 ± 0.01 ***b***	6.2 ± 0.1 ***b***
	+C	3.83 ± 0.32	20.84 ± 4.23	294 ± 50.51	0.66 ± 0.21	38.37 ± 0.5	33.64 ± 0.53	27.98 ± 0.83	0.77 ± 0.01	6.18 ± 0.11
	+P	3.54 ± 0.36	21.83 ± 3.41	269.2 ± 39.43	22.37 ± 1.48*	39.24 ± 0.47	34.01 ± 0.42	26.75 ± 0.79	0.75 ± 0.01	6.2 ± 0.11
	+C+P	3.40 ± 0.21	37.45 ± 7.73	311.4 ± 41.85	20.26 ± 1.81*	38.03 ± 0.67	32.77 ± 0.68	29.21 ± 1.27	0.75 ± 0.01	5.88 ± 0.21*
EOM	Control	3.47 ± 0.09 **a**	73.23 ± 4.02 ***a***	368.7 ± 42.81 ***a***	0.69 ± 0.19 ***a***	43.04 ± 0.27 ***a***	39.25 ± 0.33 ***a***	17.7 ± 0.34 ***b***	0.65 ± 0.01 ***c***	5.05 ± 0.05 ***c***
	+C	3.78 ± 0.09	87.66 ± 15.04	296.7 ± 36.47	3.48 ± 1.59	41.99 ± 0.65	38.94 ± 0.77	19.06 ± 0.68	0.65 ± 0.01	5.04 ± 0.08
	+P	3.59 ± 0.17	61.52 ± 4.29	284.4 ± 22.96	25.64 ± 1.35*	42.59 ± 0.52	39.22 ± 0.7	18.19 ± 1.1	0.65 ± 0.01	5.06 ± 0.07
	+C+P	3.81 ± 0.15	63.59 ± 8.24	304.4 ± 35.83	24.83 ± 2.1*	43.53 ± 0.25	39.37 ± 0.39	17.1 ± 0.3	0.64 ± 0.01	4.97 ± 0.1

Seven parallel factor analysis components (PCs) describing the quality of OM pools were identified from the model, and their spectral properties were compared with those of Stedmon and Markager (2005) (Table 5, Figure S3 and Table S3). PC2 and PC4, as well as PC1 and PC3 in Raman units were pooled for humic‐like and fulvic acid‐like OM pools, respectively; tryptophan‐like PC5, tyrosine‐like PC7, and an unknown free amino acid PC6 (Ex 300/ Em 338, Murphy, Ruiz, Dunsmuir, & Waite, 2006) were treated as the protein‐like pool (Table 5). Organic substrate used in the experiment was the only factor influencing the percentage of each OM pool (Table 4). When comparing with LAKE_control_, BOM_control_ yielded a higher percentage of the humic‐like fluorophore pool, a lower fulvic‐like pool, but a similar percentage of protein‐like pool. EOM_control_ had high humic and fulvic‐like pools, but a lower protein‐like pool than LAKE_control_ (Table 4). As indicated by a low E_2_: E_3_ and BIX, both BOM_control_ and EOM_control_ had higher mean molecular weight and higher recalcitrance than LAKE_control_ (*p* < 0.001); however, the molecular weight and recalcitrance of EOM was also higher than BOM. BOM_+C+P_ had significantly higher mean molecular weight than BOM_control_. Based on the SMA regression, the mean molecular weight increased with the fluorescence intensity of the humic‐pool in EOM regardless of nutrient addition (*n* = 84, *r* ^2^ = 0.23, *p* < 0.001), but this relationship was absent for BOM. Furthermore, both mean molecular weight and humic‐pool had negative relationships with BIX in EOM substrate (*n* = 84, *r* ^2^ = 0.19, *p* < 0.001), but this relationship was rather weak for BOM (*n* = 84, *r* ^2^ = 0.08, *p* = 0.007).

**TABLE 5:** Properties of the seven parallel factor analysis components (PCs) identified from the fluorescent dissolved organic matter of this study

Components Stedmon and Markager (2005)	This study	Excitation and emission maxima	Origin	Properties
PC1	PC4	Ex < 250/Em 448	Terrestrial	Humic fluorophore group
PC3	PC2	Ex < 250/Em 412	Terrestrial	Humic fluorophore group
PC2	PC3	Ex < 250/Em 504	Terrestrial/autochthonous	Fulvic acid fluorophore group
PC4	PC1	Ex < 250/Em 440	Terrestrial/autochthonous	Fulvic acid fluorophore group
PC7	PC5	Ex 280/Em 344	Autochthonous	Tryptophan fluorophore (protein)
PC8	PC7	Ex 275/Em 304	Autochthonous	Tyrosine fluorophore (protein)

### 3.3 Bacterioplankton metabolism

**FIGURE 1:**
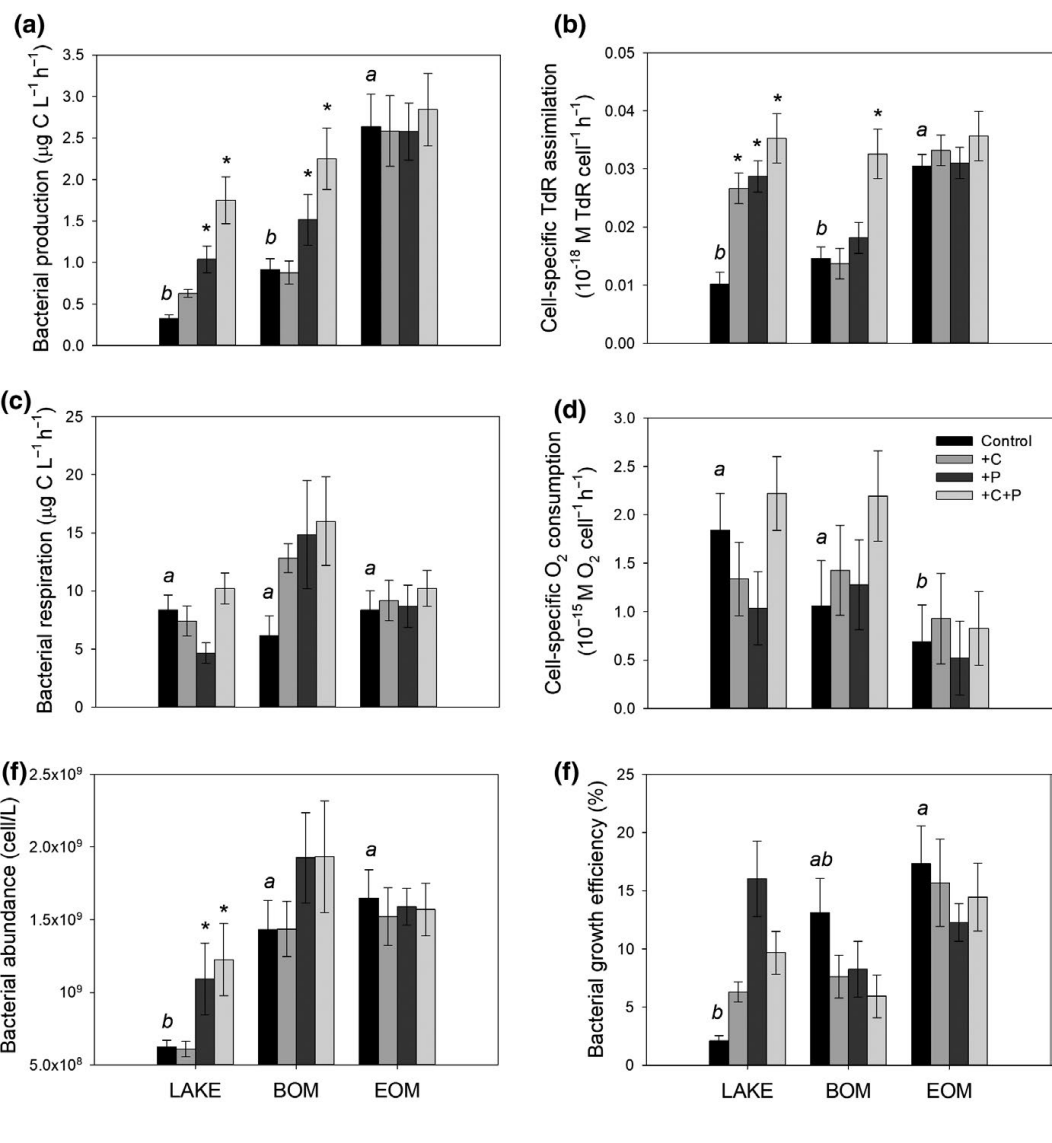
Bacterioplankton metabolism: (a) bacterial production; (b) cell‐specific TdR assimilation; (c) bacterial respiration; (d) cell‐specific O_2_ consumption; (e) bacterial abundance; and (f) bacterial growth efficiency of Lake water (LAKE), bamboo and evergreen forest terrigenous organic matter (BOM and EOM, respectively). Each OM substrate had control, +C, +P, and +C+P nutrient addition treatments which were distinguished by bar colours shown in the legend. Letters mark the significant differences (*p* < 0.05) between tOM substrate and LAKE_control_; asterisks mark the significant differences between nutrient addition and the respective OM substrate control (rmANOVA, Means ± 1 *SEM*, *n* = 21)

**FIGURE 2:**
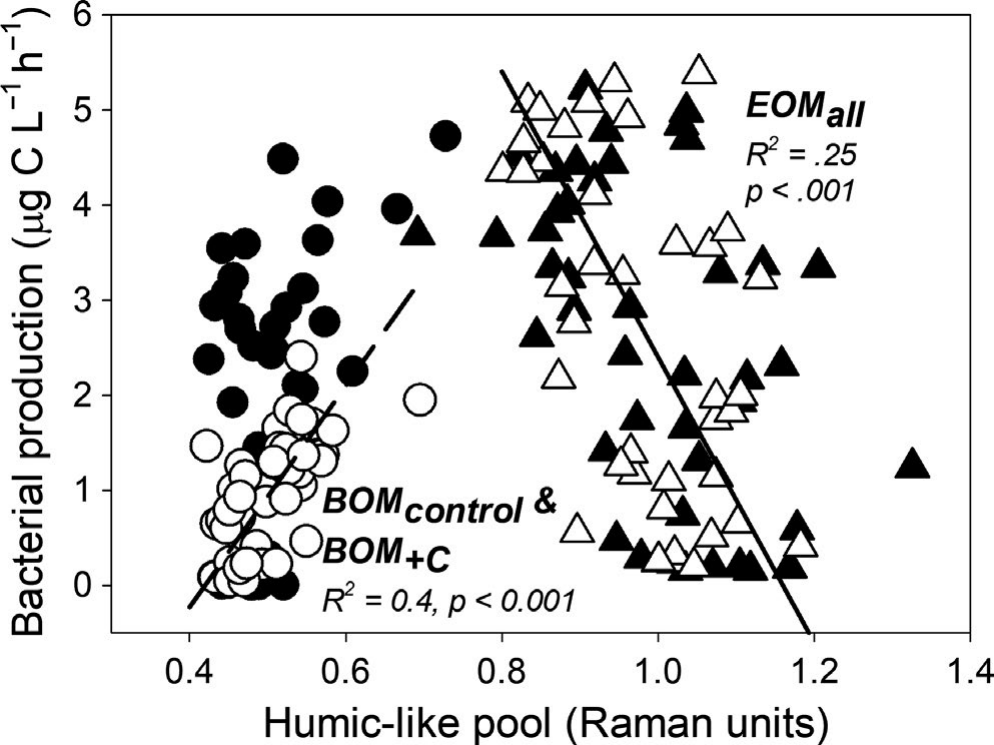
Bacterial production versus humic‐like OM pool. Bamboo (circles and dashed regression line, *n* = 84) and evergreen forest terrigenous organic matter (triangles and solid line, *n* = 84). Open symbols = samples without P addition; black symbols = samples with P addition

Bacterial production was significantly higher in EOM_control_ than LAKE_control_ (*p* < 0.001, Figure 1a). The BP of +P and +C+P treatment of LAKE and BOM were significantly higher than those of LAKE_control_ and BOM_control_, respectively. EOM_control_ had the highest cell‐specific TdR assimilation rate, while BOM_control_ had similar value as LAKE_control_ (Figure 1b). However, all nutrient treatments of LAKE and BOM_+C+P_ showed higher cell‐specific TdR assimilation rate compared to LAKE_control_ and BOM_control_, respectively. Bacterial production was positively related to BIX—the freshly released dissolved OM (SMA, *r* ^2^ = 0.38, *p* < 0.001, slope = 10.2), the fluorescence intensity of humic and fulvic‐like pools (*r* ^2^ = 0.4 and 0.2, *p* < 0.001 and *p* = 0.015, slope = 16.9 and 9.13) in BOM_control_ and BOM_+C_ (Figure 2); these relationships disappeared upon P addition. Moreover, BP was inversely related to the fluorescence intensity of the humic‐like pool in EOM substrate regardless of nutrient addition (*r* ^2^ = 0.26, *p* < 0.001, slope = −7.3).

Bacterial respiration was similar across different OM substrates (Figure 1c). Although nutrient addition had the most apparent effect on BOM, the differences between treatments and the control were not significant due to large variations between replicates. The cell‐specific O_2_ consumption was regulated only by OM substrate (*p* = 0.013); i.e. EOM_control_ was lower than LAKE_control_ (Figure 1d). LAKE and BOM especially had very high cell‐specific respiration in the beginning of the experiment (0.042 ± 0.008 and 0.04 ± 0.018 pg C cell^−1^ day^−1^, Figure S4). bacterial abundance was significantly enhanced by BOM and EOM substrates (*p* < 0.001, Figure 1e). The +P and +C+P treatments significantly increased cell abundance in LAKE.

**FIGURE 3:**
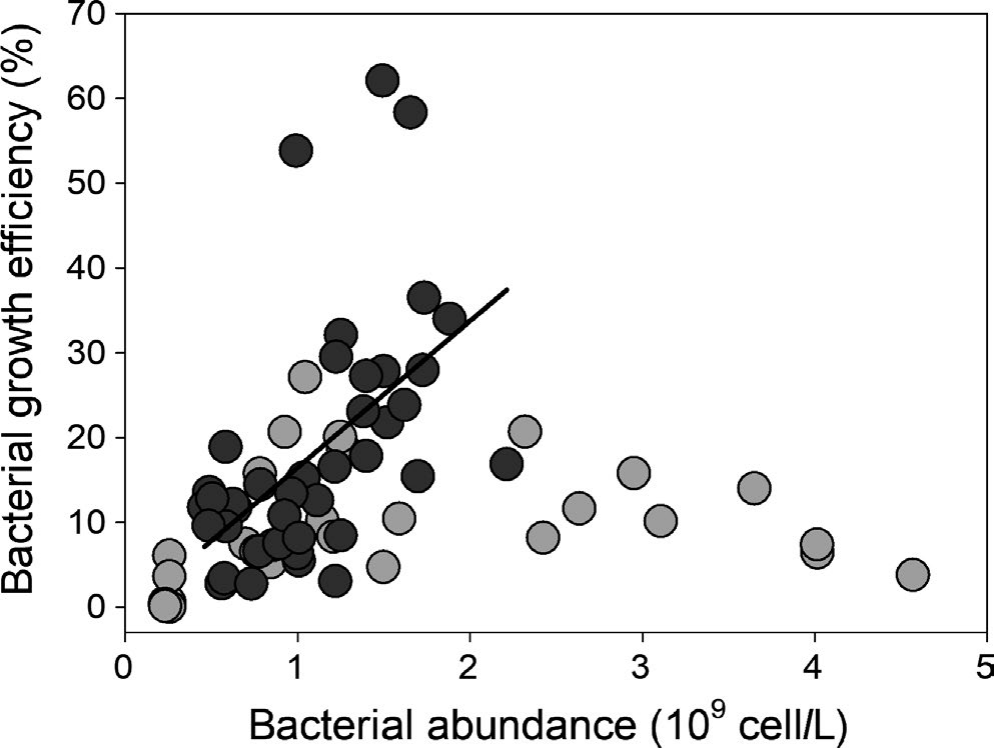
Bacterial growth efficiency versus bacterial abundance. Bamboo (light grey circles, *n* = 32) and evergreen forest terrigenous organic matter (dark grey circles and solid regression line, *n* = 44)

Bacterial growth efficiency was only influenced by OM substrate but not by nutrient addition (*p* = 0.025, Figure 1f). Evergreen OM had the highest averaged (15 ± 9.2%) and maximum growth efficiency (62%) while LAKE and BOM had lower means (9.96 ± 10.17% and 10.2 ± 8.81%, respectively). When EOM was the main substrate, growth efficiency increased with BA (*r* ^2^ = 0.35, *p* < 0.001, Figure 3). For BOM, the positive growth efficiency versus cell number correlation only held until growth efficiency reached about 20%; the relationship became negative as BA reached its maximum.

### 3.4 Enzymatic activity

**FIGURE 4:**
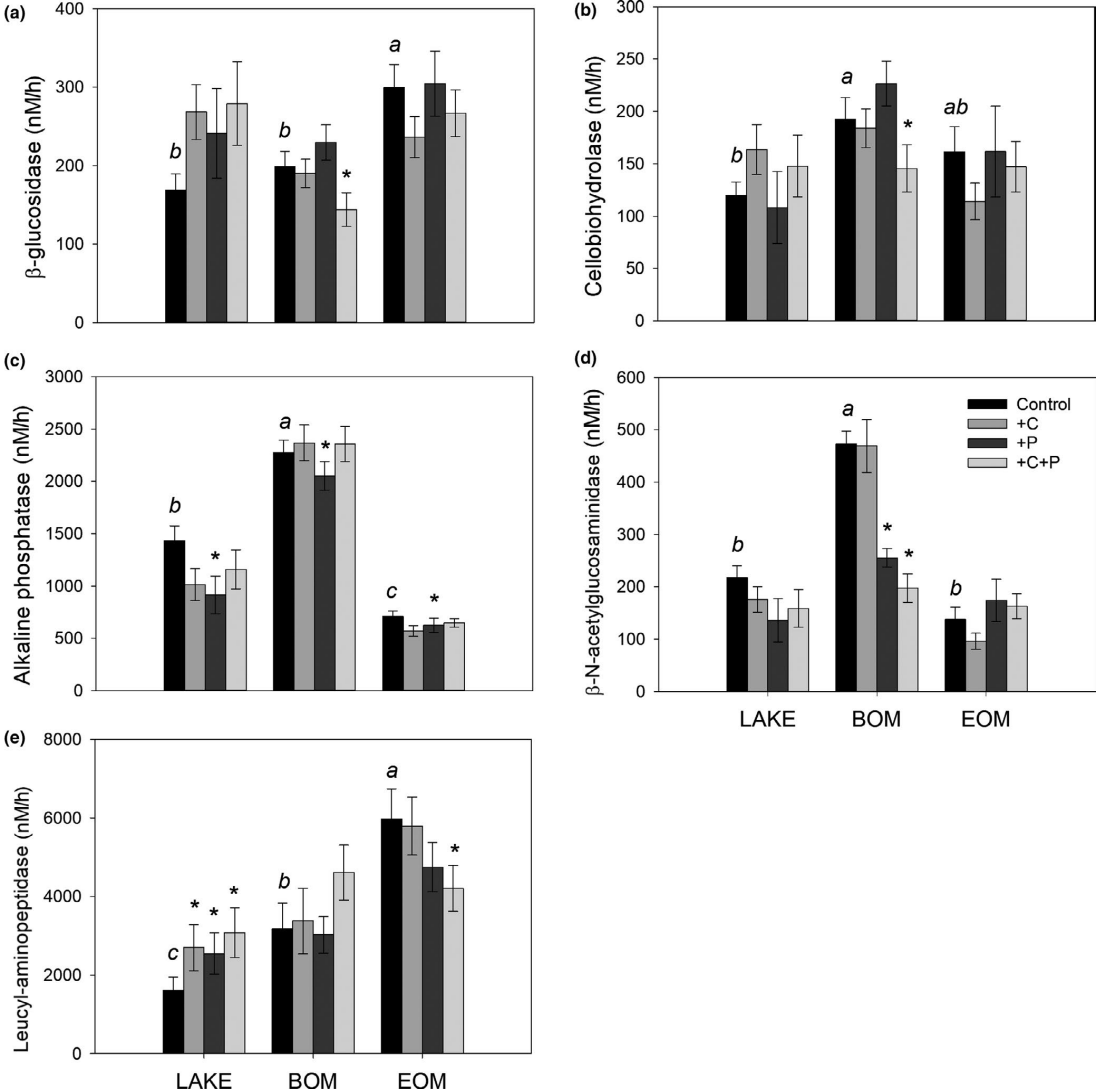
Exoenzyme activity: (a) β‐glucosidase (BG); (b) cellobiohydrolase (CBH); (c) alkaline phosphatase (AP); (d) β‐N‐acetylglucosaminidase (NAG); and (e) leucyl‐aminopeptidase (LAP) of Lake water (LAKE), bamboo and evergreen forest terrigenous organic matter (BOM and EOM, respectively). Each substrate had control, +C, +P, and +C+P nutrient addition treatment (bar colours shown in legend). Letters mark the significant differences between tOM substrate and LAKE_control_; asterisks mark the significant differences between nutrient additions and the respective OM substrate control (rmANOVA, Means ± 1 *SEM*, *n* = 21)

Carbon‐hydrolysing enzymes BG activity in EOM_control_ and cellobiohydrolase (CBH) activity in BOM_control_ were significantly higher than LAKE_control_ (*p* = 0.001 and 0.017, respectively, Figure 4a,b). BOM_+C+P_ had significantly lower BG and CBH activity than BOM_control_ (*p* = 0.002 and 0.002, respectively).

Phosphorous‐hydrolysing enzyme alkaline phosphatase activity in BOM was significantly higher than LAKE_control_ regardless of nutrient addition (*p* < 0.001). The +P treatment of each OM substrate had significantly lower activity than their respective controls (*p* = 0.015, Figure 4c).

Nitrogen‐hydrolysing enzymes β‐N‐acetylglucosaminidase (NAG) activity in BOM_control_ and leucyl‐aminopeptidase (LAP) activity in BOM_control_ and EOM_control_ were significantly higher than for LAKE_control_ (*p* < 0.001, Figure 4d,e). Moreover, BOM_+P_ and BOM_+C+P_ had lower NAG activity than BOM_control_ (*p* < 0.001). Leucyl‐aminopeptidase activity increased in all nutrient additions when LAKE was the only OM substrate (*p* < 0.001, Figure 4e). However, LAP activity was significantly lower in EOM_+C+P_ than EOM_control_ (*p* = 0.006).

### 3.5 Relationships between hydrolysing exoenzyme activities

**FIGURE 5:**
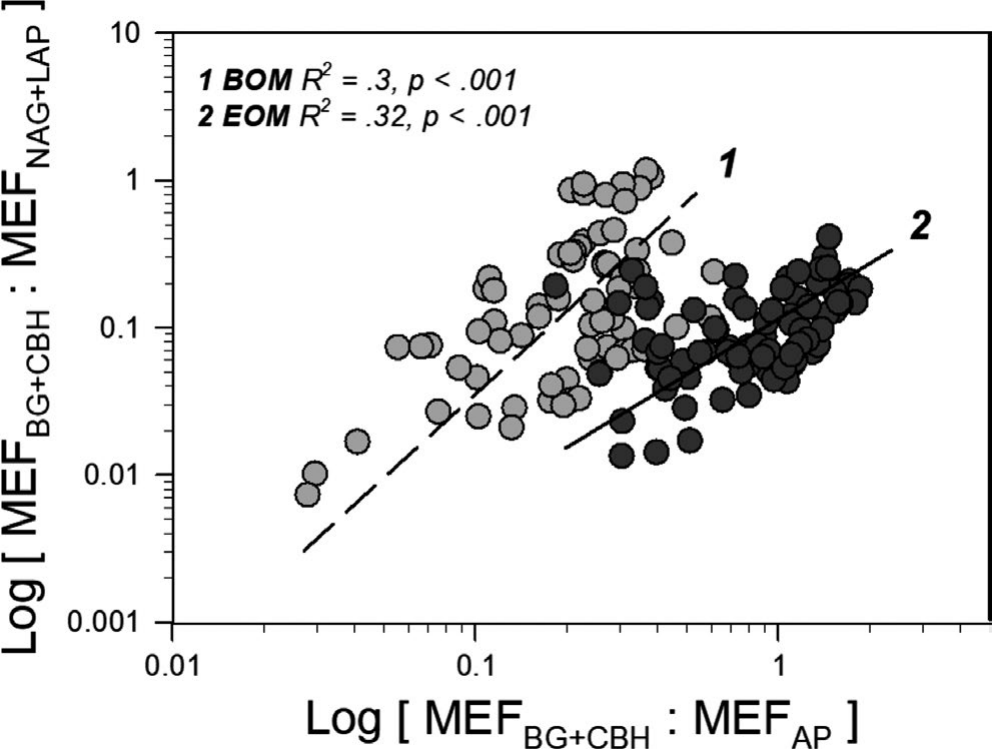
Log‐transformed C:N versus log‐transformed C:P enzyme activity ratios. BG = β‐glucosidase; CBH = cellobiohydrolase; NAG = β‐N‐acetylglucosaminidase; and LAP = leucyl‐aminopeptidase. Bamboo soil organic matter (BOM) = light‐grey circles and dashed Standardised major axis regression line, *n* = 75; and evergreen forest soil organic matter (EOM) = dark‐grey circles and solid line, *n* = 83

Using slopes of SMA regression, the scaling of MEF of C‐hydrolysing enzymes MEF_BG+CBH_ versus MEF_NAG_ or MEF_AP_ between BOM and EOM substrate was compared, as well as the difference between tOM substrate control and its nutrient additions. Significant relationships are presented in Table 6. Bamboo OM and EOM had significantly different slopes for MEF_BG+CBH_ versus MEF_AP_ and MEF_BG+CBH_ versus MEF_NAG_ relationships (*p* = 0.008 and <0.001, respectively) but similar slopes for MEF_BG+CBH_ versus MEF_LAP_ (*p* = 0.901). BOM yielded significantly higher slopes of both enzyme‐scaling relationships than EOM. Nutrient addition only had effects on BOM substrate in terms of N and P acquisition scaling; i.e. the slope of MEF_BG+CBH_ versus MEF_AP_ for BOM_+C+P_ was significantly lower than that of the BOM_control_, and the slope of MEF_BG+CBH_ versus MEF_NAG_ was lower for BOM_+P_ and BOM_+C+P_ than for BOM_control_. Standardised major axis regression between potential exoenzyme activity C:N versus C:P, denoted the relationship between activities of N‐ and P‐hydrolase relative to C‐acquisition (Figure 5), with OM substrate being the only factor regulating the slopes. Significantly different slopes for BOM and EOM (slope = 2.03 and 1.34, respectively, *p* = 0.002) were observed; however, this SMA regression was not significant for LAKE substrate.

**TABLE 6:** Significantly different standardised major axis slopes between metabolic effort of MEF_BG+CBH_ versus MEF_NAG_ or MEF_AP_. Italic and bold letters denote these statistical differences in slopes at a significant level of *p* < 0.05

Enzyme MEF	Treatment	*n*	*r* ^2^	Slope	95% CI	Intercept
MEF_BG+CBH_ versus MEF_AP_	LAKE_control_	41	0.5	1.04 ***ab***	0.83–1.31	−0.74
	BOM_control_	20	0.73	1.52 ***a***	1.18–1.96	−1.08
	EOM_control_	21	0.72	0.96 ***b***	0.74–1.24	−0.25
MEF_BG+CBH_ versus MEF_NAG_	LAKE_control_	41	0.63	1.05 ***b***	0.86–1.27	0.14
	BOM_control_	20	0.84	1.74 ***a***	1.43–2.12	−0.01
	EOM_control_	20	0.72	0.87 ***b***	0.67–1.13	0.45
MEF_BG+CBH_ versus MEF_AP_	BOM_control_	20	0.77	1.44 ***a***	1.14–1.82	−1.32
	BOM_+C+P_	16	0.98	0.98 ***b***	0.9–1.06	−1.13
MEF_BG+CBH_ versus MEF_NAG_	BOM_control_	20	0.86	1.65 ***a***	1.37–1.98	−0.29
	BOM_+P_	20	0.99	1.08 ***b***	1.02–1.14	−0.03
	BOM_+C+P_	16	0.99	1.04 ***b***	1–1.09	−0.11

Abbreviations: AP, alkaline phosphatase; BG, β‐glucosidase; BOM & EOM, bamboo‐ and evergreen forest soil‐derived dissolved organic matter, respectively; CBH, cellobiohydrolase; NAG, β‐N‐acetylglucosaminidase.

### 3.6 Constrained RDA

The +C treatments were omitted from the analysis due to an insignificant contribution to the model. The constrained variables (*BOM*,* EOM*, and +*P*) explained 42% of total variance; the unconstrained variable accounted for 49.7% and *Time* accounted for 8.3% of total variance. The first and the second RDA axes accounted for 51.2 and 29% of total variance, respectively. The permutation test result of observed values was not different from that of the permutation on *Time*, implying there was a significant linear relationship between the response matrix and the explanatory matrix.

**FIGURE 6:**
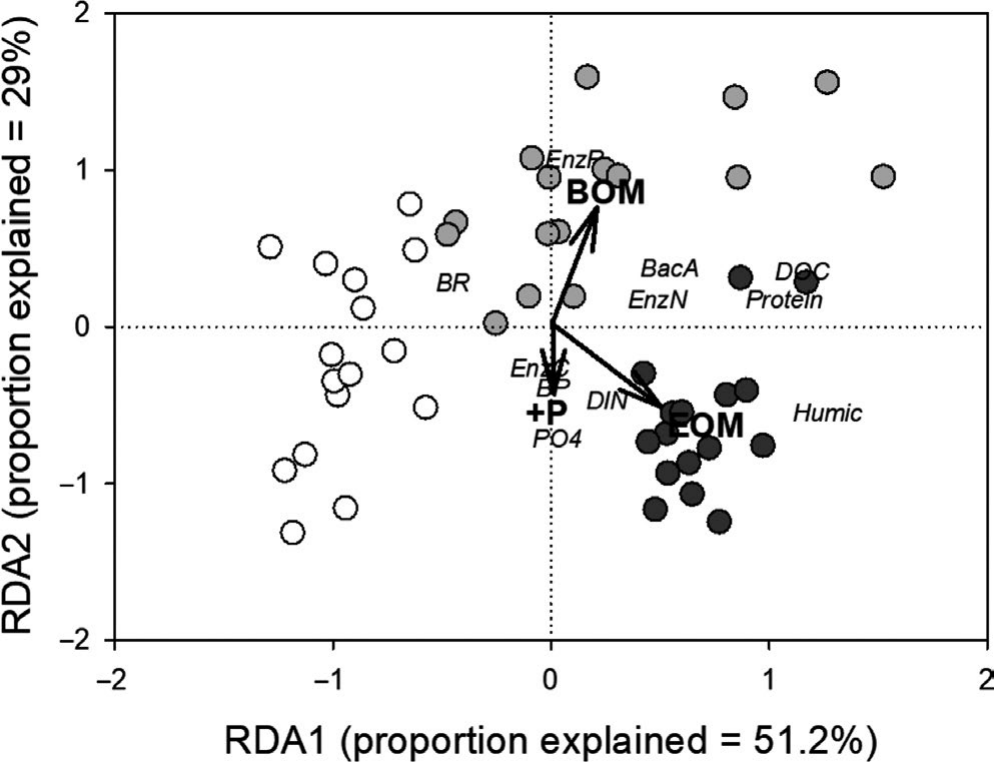
Constrained redundancy analysis (RDA) of water chemistry and bacterial metabolic data from experiment day 0, 4, 7, and 10 (*N* = 48). Lake water control (white circles, *n* = 16); Environmental variables (black arrows with bold font) using binary data: BOM = bamboo soil organic matter (light grey circles, *n* = 16); EOM = evergreen forest soil organic matter (dark grey circles, *n* = 16); +P = P addition. Response variables in italic: DOC = dissolved organic carbon; DIN = dissolved inorganic nitrogen; PO4 = phosphate; BacA = bacterial abundance; BP = bacterial secondary production; BR = bacterial respiration. Enzyme activity: EnzC = β‐glucosidase and cellobiohydrolase; EnzN = β‐N‐acetylglucosaminidase and leucyl‐aminopeptidase; EnzP = alkaline phosphatase; fluorescent dissolved organic matter—humic and protein‐like property

The first RDA axis was mainly attributed to the presence of tOM, separating BOM and EOM from LAKE (Figure 6). The second axis defined the effect of P concentration, separating the higher P content EOM and P‐addition treatments from BOM. Both tOM substrates were associated with DOC concentration, N‐hydrolysing enzymes, protein‐like OM, and BA. However, EOM substrate was also associated with DIN concentration and humic‐like OM pool, while BOM was related to P‐hydrolysing enzyme. LAKE samples were only associated with BR. The +P addition was related to phosphate concentration, BP, and C‐hydrolysing enzyme activity. In sum, tOM addition was the strongest factor controlling data ordination. In comparison to EOM, microbes using BOM as the main substrate were possibly subjected to dramatic changes when receiving additional P, as seen from the high variation between BOM and the +P treatment occupying opposite sides of the second axis.

## 4 DISCUSSION

In this study, we investigated the effects of OC lability and P availability of tOM on aquatic bacterioplankton metabolic strategies. We found that the two tOM conditions induced different metabolic pathways. Differences in the enzymatic activities and their ratios show that this disparity is probably the result of nutrient availability rather than OC lability, and that the bacteria assemblages strategically targeted different organic compounds (e.g. Clinton et al., 2010).

### 4.1 Effects of terrigenous substrates and additional labile OC on bacterioplankton metabolism

Substrate availability is a main factor that regulates bacterial growth efficiency—i.e. the percentage of carbon assimilated into biomass of the total amount of carbon consumed (Middelboe & Søndergaard, 1993). Bamboo forest soil OM had a lower percentage of N and P, which was associated with lower BP at the cellular level. However, this was compensated by higher cell abundance, ultimately resulting in a bacterial growth efficiency between that of LAKE and EOM. Soil studies have shown that labile carbon functional groups O‐alkyl‐C and carboxyl‐C, which constitute carbohydrates or cellulose in fresh plant material (Solomon et al., 2010), are significantly higher in BOM than that of woody forests (Wang et al., 2016). However, the labile carbon did not translate to higher growth efficiencies. The BOM_control_ treatment did not exhibit a higher degree of cell‐specific TdR assimilation, cell‐specific O_2_ consumption rates, and bacterial growth efficiencies compared to that of LAKE_control_. Bamboo OM might support increased BA but not production because the substrate has limited nutrition values, especially in P. This limitation can impact the regulation of bacterial physiology and metabolism. For instance, it has been shown that microbes may reduce the size of their DNA pool by maintaining fewer genome copies under P‐limited conditions (Zerulla et al., 2014). We observed that BOM treatments induced exoenzymatic activities associated with polymer breakdown, which are often inversely correlated to bacterial growth efficiency (Middelboe & Søndergaard, 1993).

The EOM_control_ treatment had significantly higher increase of BP and showed the highest mean molecular weight and yielded the highest percentage of humic‐ and fulvic acid‐like pool but with the lowest percentage of protein‐like compounds. The humic‐like OM pool and mean molecular weight of EOM_control_ were negatively correlated to biological index and BP. This agreed with the general model that the degree of substrate recalcitrance, in terms of microbial degradation, is positively correlated with the molecular weight of the OM pool (Saunders, 1976). This complex substrate induced the BG and leucyl‐aminopeptidase activities in EOM_control_, the C and N released from the substrate were used for higher BP (Foreman et al., 1998). The metabolic activity of EOM_control_ is in line with studies demonstrating that, under low concentrations of labile C, there is a bacterial preference for humic‐like compounds over dissolved inorganic nitrogen as the former is a source of both C and N (Carlsson, Segatto, & Granéli, 1993; Ghosh & Leff, 2013).

When there is a surplus of carbon relative to available nutrients, bacteria often dispose of the excess carbon to help maintain intracellular stoichiometry. This is done by increasing respiration or by excreting extracellular polysaccharides or metabolites to dissipate extra energy (Decho, 1990; Hessen & Anderson, 2008; Linton, 1990). In this study, additional acetate was used to test the OC limitation when bacterioplankton uses the two tOM sources. Although not statistically significant, cell‐specific O_2_ consumption rates increased in both tOM substrates receiving + C or +C+P, whereas in LAKE conditions there was no such trend. This increase could potentially be attributed to an *priming effect*, that the added labile OC influenced the mineralisation rate of relatively recalcitrant tOM (Guenet et al., 2014). However, only the LAKE_+C_ treatment, not BOM_+C_ or EOM_+C,_ induced cell‐specific TdR incorporation rates. Our result reflected imbalanced ambient inorganic nutrients in BOM_+C_ for bacteria to incorporate the added carbon. The added acetate was probably respired efficiently, leading to an 108% increase in respiration rate in BOM_+C_ without inducing other metabolic responses. However, this increase was not statistically significant due to large variability in respiration measurements. Although acetate provides a labile OC source, it is a relatively oxidised organic substrate poor in energy and may instead trigger the microbial community to maintain respiration or produce enzymes (del Giorgio & Cole, 1998).

By contrast, EOM_+C_ showed a slight reduction of BG and CBH activities by 21 and 29%, respectively, when compared to EOM_control_, suggesting that bacteria may have used acetate as a carbon source in addition to humic substrate. However, this result was statistically not significant. The experimental design could be a part of the reason. Following Berggren, Laudon, et al. (2010), who found that acetate is an important OC source for BP, we added similar concentrations of acetate to our treatments, but much lower than that of Steen et al. (2016). Furthermore, the amount of acetate added was probably proportionally too small compared to what was released from the tOM substrates, considering that 1 g of forest soil could form about 35–220 μg acetate per 24 hr in the aquatic context (Küsel & Drake, 1995).

Although discussions on microbial life cycle and community structure exceed the scope of this study, it merits further investigation. Bacterial growth efficiency tends to decrease as substrate quality (in terms of nutrient content) drops and population generation time increases (Middelboe & Søndergaard, 1993). On the one hand, HMW compounds might support taxa that can produce certain enzymes. This could reduce bacterial diversity upon HMW compound addition (Balmonte et al., 2019). On the other hand, however, some have suggested that humic substances may instead promote microbial diversity, as the enzymatic breakdown of complex substrates can support members in the community (Ghosh & Leff, 2013).

Overall, our result echoes the observation by Guillemette et al. (2016) that bacterioplankton use different metabolic pathways when using labile versus complex carbon. The more labile substrate was preferentially allocated to maintain respiration and exoenzyme production, whereas complex and recalcitrant tOM was preferentially allocated to BP and the carbon was repackaged into biomass. However, the acetate experiments suggested that nutrients might be the more important factor causing this disparity rather than OC lability between the two types of tOM. Indeed, the nutrition‐rich EOM substrate induced greater bacterial growth efficiency regardless of further acetate or phosphorus treatments, with significantly higher TdR incorporation rates and lower O_2_ consumption rates at a cellular level.

### 4.2 Effect of additional inorganic phosphorus on bacterial degradation of terrigenous substrates

The difference in soil tOM quality is derived from vegetation type. In particular, the P content of tOM is a major parameter influencing the production of freshwater bacterioplankton in the receiving waters (Lennon & Pfaff, 2005). Terrigenous OM contains organic P, which could alleviates P limitation of bacterioplankton production (Soares et al., 2018). The increase of BA is often the primary response when phosphorus‐limited bacteria receive an input of P (Middelboe, Jørgensen, & Kroer, 1996; Steen et al., 2016). In our study, this was especially true for the EOM which showed significant increase in both BP and abundance with or without inorganic P addition. The organic P source in BOM_control_ and EOM_control_ increased the bacterial cell numbers (129 and 164% higher than that of LAKE_control_ on average, respectively). Under P‐limitation, the uptake of C in BOM_control_ and BOM_+C_ was allocated to respiration to maintain cellular functions such as membrane integrity, active transport systems, nutrient acquisition, and enzyme production (del Giorgio & Cole, 1998; Russell, 1991). Therefore, P‐addition had a significant impact on the P‐limited BOM substrate, which yielded an significantly increased BA in BOM_+P_ and BOM_+C+P_ (35% higher than that of BOM_control_) and an increased trend of BA. This suggested that the bacteria switched to use inorganic phosphate in these treatments, as bacteria production disassociated from the OM properties. Since inorganic nutrients are expensive energy sources, BR of OC is increased to meet the rising energy demands (Middelboe & Søndergaard, 1993). Subsequently, bacterial growth efficiency of BOM started to decline (uncoupling from BA) as it reached approximately 20%. One factor that uncouples growth rate (population generation time) from bacterial growth efficiency is that bacteria may maximise growth at the expense of efficiency (Russell, 1991).

Exoenzyme ratios of C:N and C:P are indicators of environmental CNP stoichiometry. The differences in slopes of the production‐specific exoenzyme activities—MEF_BG+CBH_/MEF_NAG+LAP_ versus MEF_BG+CBH_/MEF_AP_ relationships indicate differences in the stoichiometry of N and P acquisition relative to C availability (Sinsabaugh et al., 2010). Scaling relationships for exoenzyme activities normalised to productivity showed that the bacteria potentially acquired more units of C per acquired P when using BOM_control_. This value dropped after BOM received external P input. Moreover, the ratio between NAG and LAP signals the relative importance of the cell wall‐derived aminosaccharides such as chitin as a N source (Sinsabaugh et al., 2010). In our study, BOM_control_ and BOM_+C_ had a ratio of 0.5, which was 18.5 times higher than that of EOM_control_ and EOM_+C_ (ratio = 0.027). This shows that the breakdown of peptidoglycan in bacterial cell walls (Benner & Kaiser, 2003) or fungal chitin (Gooday, 1990) was relatively important in BOM. Bacterial breakdown of aminosaccharides in turn generated sources of N such as small oligosaccharides and N‐acetylglucosamine, which can be directly incorporated into bacterial cells (Wetzel, 1991). This was supported by the significant positive correlation between BP and the fresh released OM pool indicated by BIX in BOM_control_ and BOM_+C_ treatments. Moreover, bacteria probably used the released fresh dissolved OM to produce humic‐like bacterial substances (e.g. Guillemette & del Giorgio, 2012), as the fluorescence intensity of humic‐like pool of these treatments increased along the BP gradient. The concentration of N‐acetylglucosamine also promotes the chitinase gene expression of chitinolytic bacteria (Delpin & Goodman, 2009), potentially leading to the rapid turnover of this specific bacterial assemblage that preferentially uses labile BOM (e.g. Crump, Kling, Bahr, & Hobbie, 2003). The chitinolytic degradation process could have generated deacetylated cellulose‐like molecules (Beier & Bertilsson, 2013) and contributed to the simultaneous increase of CBH activity in the BOM_control_. However, the advantage of using aminosaccharides to compensate N requirement decreased after BOM treatments received inorganic P (NAG to LAP ratio = 0.1).

EOM, by contrast, provided 87.6% more dissolved organic P than BOM. Using this organic P source may have allowed bacteria to also utilise more recalcitrant carbon molecules (Benner, Lay, K&nees, & Hodson, 1988), like humic substances, probably through enhanced exoenzyme production (Sinsabaugh, Findlay, Franchini, & Fischer, 1997). However, phosphatase activity often negatively correlates to inorganic P concentration (Clinton et al., 2010), as seen in the decreased phosphatase activity in EOM treatment and all +P treatments regardless of tOM source. As the bacteria in EOM_+P_ and EOM_+C+P_ also consumed the added inorganic phosphate for production, it is likely that the P demand in these treatments increased. As the cell number and production in EOM_+P_ and EOM_+C+P_ remained similar to that of EOM_control_, the rising P‐demand was probably related to the need to synthesise more phosphorus‐rich molecules (Yao et al., 2016).

The RDA sums up the interactive effect between tOM, additional acetate, and phosphate on aquatic bacterioplankton metabolism. We argue that EOM provided a nutrient‐ and humic‐rich tOM substrate, which had a higher capacity to maintain metabolic direction towards bacteria production and yield higher bacterial growth efficiency. This was similar to the results of previous experiments that added aged humic material to bacterioplankton assemblages (e.g. Eiler, Langenheder, Bertilsson, & Tranvik, 2003; Lennon & Pfaff, 2005). Humic‐rich EOM stimulated BG activity to produce available OC. However, with the acetate concentration we added in this experiment, labile OC proved to be a statistically insignificant term in the RDA model. Both LAKE and BOM allocated most resources into activities related to molecular breakdown and were more influenced by additional phosphorus. bamboo OM provided a labile OC source that enhanced N‐ and P‐hydrolysing enzyme activities. Once they received additional P, bacterioplankton dramatically changed either their metabolic strategy or community structure to maintain their stoichiometric requirements.

## 5 CONCLUSION

Our results show that BOM and EOM provide bacterioplankton with substrates of different OM quality and P content, inducing distinct metabolic pathways (BP or respiration). These results have several important implications for future research.

We showed that bacterioplankton in BOM treatments invested in cell numbers and exoenzyme activities that hydrolyse aminosaccharides, which could be associated with the breakdown of microbial detritus (e.g. cell walls) in the tOM pool. The metabolism of microbially‐derived carbon may thus unlock carbon sequestrated in microbial biomass of terrigenous carbon sources in aquatic systems. Future research can further investigate whether microbially‐derived or plant‐derived compounds were consumed for bacterial growth or respiration with, for instance, tracer techniques.

Another implication is that aquatic systems dominated by BOM inputs are likely to increase their bacterial cell numbers, produce exoenzymes, and respire more C after receiving abundant terrigenous phosphorus eroded seasonally during heavy rainfall events in steep riparian zones (Lee et al., 2013). Furthermore, as tillage and N fertilisers are commonly used in bamboo forests for enhancing vegetation biomass production (He & Ye, 1987), N residues entering the aquatic systems will be likely to change the nutrient acquisition strategy of aquatic microbial assemblages. This is because there is interdependence between the acquisition of organic N and P in microbial communities in BOM as opposed to EOM substrates. We thus advise future research to focus on the effect of N manipulation on aquatic metabolism using BOM as the main substrate.

Finally, as tOM substrates originating from different vegetation types can induce distinct bacterioplankton metabolic pathways, future model‐building should take tOM quality into consideration when estimating soil carbon turnover rates along a terrestrial–aquatic continuum.

## CONFLICT OF INTEREST

The authors declare no conflict of interest.

## Supporting information

Supplementary Material

Supplementary Material

## Data Availability

Further data that support the findings of this study are available from the corresponding author upon reasonable request.

## References

[fwb13593-bib-0001] Balmonte, J. B. , Buckley, A. , Hoarfrost, A. , Ghobrial, S. , Ziervogel, K. , Teske, A. , & Arnosti, C. (2019). Community structural differences shape microbial responses to high molecular weight organic matter. Environmental Microbiology, 21, 557–571. 10.1111/1462-2920.14485 30452115

[fwb13593-bib-0002] Beier, S. , & Bertilsson, S. (2013). Bacterial chitin degradation—Mechanisms and ecophysiological strategies. Frontiers in Microbiology, 4, 149 10.3389/fmicb.2013.00149 23785358 PMC3682446

[fwb13593-bib-0003] Benner, R. , & Kaiser, K. (2003). Abundance of amino sugars and peptidoglycan in marine particulate and dissolved organic matter. Limnology and Oceanography, 48, 118–128. 10.4319/lo.2003.48.1.0118

[fwb13593-bib-0004] Benner, R. , Lay, J. , K&nees, E. , & Hodson, R. E. (1988). Carbon conversion efficiency for bacterial growth on lignocellulose: Implications for detritus‐based food webs. Limnology and Oceanography, 33(6), 1514–1526. 10.4319/lo.1988.33.6part2.1514

[fwb13593-bib-0005] Berggren, M. , Laudon, H. , Haei, M. , Ström, L. , & Jansson, M. (2010). Efficient aquatic bacterial metabolism of dissolved low‐molecular‐weight compounds from terrestrial sources. The ISME Journal, 4, 408–416. 10.1038/ismej.2009.120 19907505

[fwb13593-bib-0006] Berggren, M. , Ström, L. , Laudon, H. , Karlsson, J. , Jonsson, A. , Giesler, R. , … Jansson, M. (2010). Lake secondary production fueled by rapid transfer of low molecular weight organic carbon from terrestrial sources to aquatic consumers. Ecology Letters, 13, 870–880. 10.1111/j.1461-0248.2010.01483.x 20482576

[fwb13593-bib-0007] Carlsson, P. , Segatto, A. Z. , & Granéli, E. (1993). Nitrogen bound to humic matter of terrestrial origin—A nitrogen pool for coastal phytoplankton? Marine Ecology Progress Series, 97, 105–116. 10.3354/meps097105

[fwb13593-bib-0008] Carreiro, M. M. , Sinsabaugh, R. L. , Repert, D. A. , & Parkhurst, D. F. (2000). Microbial enzyme shifts explain litter decay responses to simulated nitrogen deposition. Ecology, 81, 2359–2365. 10.1890/0012-9658(2000)081[2359:MESELD]2.0.CO;2

[fwb13593-bib-0009] Chang, E. H. , & Chiu, C. Y. (2015). Changes in soil microbial community structure and activity in a cedar plantation invaded by moso bamboo. Applied Soil Ecology, 91, 1–7. 10.1016/j.apsoil.2015.02.001

[fwb13593-bib-0010] Cho, B. C. , & Azam, F. (1988). Major role of bacteria in biogeochemical fluxes in the ocean’s interior. Nature, 332, 441–443. 10.1038/332441a0

[fwb13593-bib-0011] Chróst, R. H. (1990). Microbial ectoenzymes in aquatic environments In Overbeck, J. , & Chróst, R. J. (Eds.), Aquatic microbial ecology: Biochemical and molecular approaches (pp. 47–78). Berlin, Germany: Springer‐Verlag.

[fwb13593-bib-0012] Clinton, S. M. , Edwards, R. T. , & Findlay, S. E. G. (2010). Exoenzyme activities as indicators of dissolved organic matter composition in the hyporheic zone of a floodplain river. Freshwater Biology, 55, 1603–1615. 10.1111/j.1365-2427.2009.02383.x

[fwb13593-bib-0013] Crump, B. C. , Kling, G. W. , Bahr, M. , & Hobbie, J. E. (2003). Bacterioplankton community shifts in an Arctic lake correlate with seasonal changes in organic matter source. Applied and Environment Microbiology, 69, 2253–2268. 10.1128/AEM.69.4.2253-2268.2003 PMC154827 12676708

[fwb13593-bib-0014] Decho, A. W. (1990). Microbial exopolymer secretions in oceanic environments. Oceanography and Marine Biology: An Annual Review, 28, 73–153.

[fwb13593-bib-0015] del Giorgio, P. A. , & Cole, J. J. (1998). Bacterial growth efficiency in natural aquatic systems. Annual Review of Ecology, Evolution, and Systematics, 29, 503–541. 10.1146/annurev.ecolsys.29.1.503

[fwb13593-bib-0016] Delpin, M. W. , & Goodman, A. E. (2009). Nitrogen regulates chitinase gene expression in a marine bacterium. ISME Journal, 3, 1064–1069. 10.1038/ismej.2009.49 19440232

[fwb13593-bib-0017] Eiler, A. , Langenheder, S. , Bertilsson, S. , & Tranvik, L. J. (2003). Heterotrophic bacterial growth efficiency and community structure at different natural organic carbon concentrations. Applied and Environment Microbiology, 69, 3701–3709. 10.1128/AEM.69.7.3701-3709.2003 PMC165184 12839735

[fwb13593-bib-0018] Foreman, C. M. , Franchini, P. , & Sinsabaugh, R. L. (1998). The trophic dynamics of riverine bacterioplankton: Relationships among substrate availability, ectoenzyme kinetics and growth. Limnology and Oceanography, 43, 1344–1352. 10.4319/lo.1998.43.6.1344

[fwb13593-bib-0019] Fuhrman, J. A. , & Azam, F. (1982). Thymidine incorporation as a measure of heterotrophic bacterioplankton production in marine surface waters: Evaluation and field results. Marine Biology, 66(2), 109–120. 10.1007/BF00397184

[fwb13593-bib-0020] Ghosh, S. , & Leff, L. G. (2013). Impacts of labile organic carbon concentration on organic and inorganic nitrogen utilization by a stream biofilm bacterial community. Applied and Environment Microbiology, 79(23), 7130–7141. 10.1128/AEM.01694-13 PMC3837758 24038688

[fwb13593-bib-0021] Gooday, G. W. (1990). The ecology of chitin degradation. Advances in Microbial Ecology, 11, 387–430.

[fwb13593-bib-0022] Guenet, B. , Danger, M. , Harrault, L. , Allard, B. , Jauset‐Alcala, M. , Bardoux, G. , … Lacroix, G. (2014). Fast mineralization of land‐born C in inland waters: First experimental evidences of aquatic priming effect. Hydrobiologia, 721, 35–44. 10.1007/s10750-013-1635-1

[fwb13593-bib-0023] Guillemette, F. , & del Giorgio, P. A. (2012). Simultaneous consumption and production of fluorescent dissolved organic matter by lake bacterioplankton. Environmental Microbiology, 14(6), 1432–1443. 10.1111/j.1462-2920.2012.02728.x 22429445

[fwb13593-bib-0024] Guillemette, F. , McCallister, L. , & del Giorgio, P. A. (2016). Selective consumption and metabolic allocation of terrestrial and algal carbon determine allochthony in lake bacteria. The ISME Journal, 10, 1373–1382. 10.1038/ismej.2015.215 26623544 PMC5029189

[fwb13593-bib-0025] He, L. M. , & Ye, Z. J. (1987). Application of multiple statistical analysis on the study of bamboo (*P. pubescens*) soils. Journal of Bamboo Research, 6(4), 28–40.

[fwb13593-bib-0026] Hessen, D. O. , & Anderson, T. R. (2008). Excess carbon in aquatic organisms and ecosystems: Physiological, ecological, and evolutionary implications. Limnology and Oceanography, 53(4), 1685–1696. 10.4319/lo.2008.53.4.1685

[fwb13593-bib-0027] Huguet, A. , Vacher, L. , Relexans, S. , Saubusse, S. , Froidefond, J. M. , & Parlanti, E. (2009). Properties of fluorescent dissolved organic matter in the Gironde Estuary. Organic Geochemistry, 40, 706–719. 10.1016/j.orggeochem.2009.03.002

[fwb13593-bib-0028] Hunt, A. P. , Parry, J. D. , & Hamilton‐Taylor, J. (2000). Further evidence of elemental composition as an indicator of the bioavailability of humic substances to bacteria. Limnology and Oceanography, 45(1), 237–241. 10.4319/lo.2000.45.1.0237

[fwb13593-bib-0029] Johnson, L. T. , & Tank, J. L. (2009). Diurnal variations in dissolved organic matter and ammonium uptake in six open‐canopy streams. Journal of the North American Benthological Society, 28(3), 694–708. 10.1899/08-107.1

[fwb13593-bib-0030] Karlsson, J. , Jansson, M. , & Jonsson, A. (2007). Respiration of allochthonous organic carbon in unproductive forest lakes determined by the Keeling plot method. Limnology and Oceanography, 52, 603–608. 10.4319/lo.2007.52.2.0603

[fwb13593-bib-0031] Kirchman, D. (2001). Measuring bacterial biomass production and growth rates from leucine incorporation in natural aquatic environments. Methods in Microbiology, 30, 227–237.

[fwb13593-bib-0032] Kritzberg, E. S. , Cole, J. J. , Pace, M. L. , Granéli, W. , & Bade, D. L. (2004). Autochthonous versus allochthonous carbon sources of bacteria: Results from whole‐lake 13C addition experiments. Limnology and Oceanography, 49, 588–596.

[fwb13593-bib-0077] Küsel, K. , & Drake, H. L. (1995). Effects of environmental parameters on the formation and turnover of acetate by forest soils. Applied and Environmental Microbiology, 61(10), 3667–3675. 16535147 10.1128/aem.61.10.3667-3675.1995 PMC1388709

[fwb13593-bib-0033] Lakowicz, J. R. (2006). Principles of fluorescence spectroscopy, 3rd ed. New York, NY: Springer.

[fwb13593-bib-0034] Lawaetz, A. J. , & Stedmon, C. A. (2009). Fluorescence intensity calibration using the Raman scatter peak. Applied Spectroscopy, 63, 936–940. 19678992 10.1366/000370209788964548

[fwb13593-bib-0035] Lee, T. Y. , Huang, J. C. , Kao, S. J. , & Tung, C. P. (2013). Temporal variation of nitrate and phosphate transport in headwater catchments: The hydrological controls and land use alteration. Biogeosciences, 10, 2617–2632. 10.5194/bg-10-2617-2013

[fwb13593-bib-0036] Lennon, J. T. , & Pfaff, L. E. (2005). Source and supply of terrestrial organic matter affects aquatic microbial metabolism. Aquatic Microbial Ecology, 39, 107–119. 10.3354/ame039107

[fwb13593-bib-0037] Linton, J. D. (1990). The relationship between metabolite production and the growth efficiency of the producing organism. FEMS Microbiology Reviews, 75, 1–18. 10.1111/j.1574-6968.1990.tb04083.x 2186758

[fwb13593-bib-0038] Linton, J. D. , & Stephenson, R. J. (1978). A preliminary study on growth yields in relation to the carbon and energy content of various organic growth substances. FEMS Microbiology Letters, 3, 95–98.

[fwb13593-bib-0039] McCallister, S. L. , & del Giorgio, P. A. (2008). Direct measurement of the d13C signature of carbon respired by bacteria in lakes: Linkages to potential carbon sources, ecosystem baseline metabolism, and CO2 fluxes. Limnology and Oceanography, 53, 1204–1216. 10.4319/lo.2008.53.4.1204

[fwb13593-bib-0040] Middelboe, M. B. , Jørgensen, N. O. G. , & Kroer, N. (1996). Effects of viruses on nutrient turnover and growth efficiency of non‐infected marine bacterioplankton. Applied and Environment Microbiology, 62, 1991–1997. 10.1128/AEM.62.6.1991-1997.1996 PMC1388872 16535334

[fwb13593-bib-0041] Middelboe, M. , & Søndergaard, M. (1993). Bacterioplankton growth yield: A close coupling to substrate lability and beta‐glucosidase activity. Applied and Environment Microbiology, 59, 3916–3921. 10.1128/aem.59.11.3916-3921.1993 PMC182549 16349094

[fwb13593-bib-0042] Mondini, C. , Cayuela, M. L. , Sanchez‐Monedero, M. A. , Roig, A. , & Brookes, P. C. (2006). Soil microbial biomass activation by trace amounts of readily available substrate. Biology and Fertility of Soils, 42, 542–549. 10.1007/s00374-005-0049-2

[fwb13593-bib-0043] Moran, M. A. , & Hodson, R. E. (1990). Bacterial production on humic and nonhumic components of dissolved organic carbon. Limnology and Oceanography, 35(8), 1744–l756. 10.4319/lo.1990.35.8.1744

[fwb13593-bib-0044] Murphy, K. R. , Ruiz, G. M. , Dunsmuir, W. T. M. , & Waite, T. D. (2006). Optimized parameters for fluorescence‐based verification of ballast water exchange by ships. Environmental Science and Technology, 40, 2357–2362. 16646474 10.1021/es0519381

[fwb13593-bib-0045] Murphy, K. R. , Stedmon, C. A. , Graeber, D. , & Bro, R. (2013). Fluorescence spectroscopy and multi‐way techniques. PARAFAC. Analytical Methods, 5(23), 6557–6566. 10.1039/c3ay41160e

[fwb13593-bib-0046] Oksanen, J. , Blanchet, F. G. , Friendly, M. , Kindt, R. , Legendre, P. , McGlinn, D. , … Wagner, H. (2019). vegan: Community Ecology Package. R package version 2.5‐4. https://CRAN.R‐project.org/package=vegan.

[fwb13593-bib-0047] Parham, J. A. , & Deng, S. P. (2000). Detection, quantification and characterization of β‐glucosaminidase activity in soil. Soil Biology & Biochemistry, 32, 1183–1190.

[fwb13593-bib-0048] Peuravuori, J. , & Pihlaja, K. (1997). Molecular size distribution and spectroscopic properties of aquatic humic substances. Analytica Chimica Acta, 337, 133–149. 10.1016/S0003-2670(96)00412-6

[fwb13593-bib-0049] Pollard, P. C. , & Moriarty, D. J. W. (1984). Validity of the tritiated thymidine method for estimating bacterial growth rates: Measurement of isotope dilution during DNA synthesis. Applied and Environment Microbiology, 48, 1076–1083. 10.1128/AEM.48.6.1076-1083.1984 PMC241689 6517579

[fwb13593-bib-0050] Regnier, P. , Friedlingstein, P. , Ciais, P. , Mackenzie, F. T. , Gruber, N. , Janssens, I. A. , … Thullner, M. (2013). Anthropogenic perturbation of the carbon fluxes from land to ocean. Nature Geoscience, 6, 597–607. 10.1038/ngeo1830

[fwb13593-bib-0051] Russell, J. B. (1991). A re‐assessment of bacterial growth efficiency: The heat production and membrane potential of *Streptococcus bovis* in batch and continuous culture. Archives of Microbiology, 155, 559–65. 10.1007/BF00245350 1953297

[fwb13593-bib-0052] Russell, J. B. (2007). The energy spilling reactions of bacteria and other organisms. Journal of Molecular Microbiology and Biotechnology, 13, 1–11. 10.1159/000103591 17693707

[fwb13593-bib-0053] Saunders, G. (1976). Decomposition in fresh water In Anderson J., & MacFadyen A. (Eds.), The role of terrestrial and aquatic organisms in decomposition processes (pp. 341–374). Oxford, UK: Blackwell.

[fwb13593-bib-0054] Schimel, J. P. , & Schaeffer, S. M. (2012). Microbial control over carbon cycling in soil. Frontiers in Microbiology, 3(348), 1–11. 10.3389/fmicb.2012.00348 23055998 PMC3458434

[fwb13593-bib-0055] Schomakers, J. , Jien, S.‐H. , Lee, T.‐Y. , Huang, J.‐C. , Hseu, Z.‐Y. , Lin, Z. L. , … Zehetner, F. (2017). Soil and biomass carbon re‐accumulation after landslide disturbances. Geomorphology, 288, 164–174. 10.1016/j.geomorph.2017.03.032 31293283 PMC6616031

[fwb13593-bib-0056] Sieczko, A. , & Peduzzi, P. (2014). Origin, enzymatic response and fate of dissolved organic matter during flood and non‐flood conditions in a river‐floodplain system of the Danube (Austria). Aquatic Sciences, 76, 115–129. 10.1007/s00027-013-0318-3 24415892 PMC3883529

[fwb13593-bib-0057] Sinsabaugh, R. L. , Findlay, S. , Franchini, P. , & Fischer, D. (1997). Enzymatic analysis of riverine bacterioplankton production. Limnology and Oceanography, 42, 29–38. 10.4319/lo.1997.42.1.0029

[fwb13593-bib-0058] Sinsabaugh, R. L. , Manzoni, S. , Moorhead, D. L. , & Richter, A. (2013). Carbon use efficiency of microbial communities: Stoichiometry, methodology and modelling. Ecology Letters, 16, 930–939. 10.1111/ele.12113 23627730

[fwb13593-bib-0059] Sinsabaugh, R. L. , Van Horn, D. J. , Shah, J. J. , & Findlay, S. (2010). Ecoenzymatic stoichiometry in relation to productivity for freshwater biofilm and plankton communities. Microbial Ecology, 60, 885–893. 10.1007/s00248-010-9696-4 20556375

[fwb13593-bib-0060] Smith, E. M. , & Prairie, Y. T. (2004). Bacterial metabolism and growth efficiency in lakes: The importance of phosphorus availability. Limnology and Oceanography, 49(1), 137–147. 10.4319/lo.2004.49.1.0137

[fwb13593-bib-0061] Soares, A. R. A. , Kritzberg, E. S. , Custelcean, I. , & Berggren, M. (2018). Bacterioplankton responses to increased organic carbon and nutrient loading in a boreal estuary—Separate and interactive effects on growth and respiration. Microbial Ecology, 76, 144–155. 10.1007/s00248-017-1115-7 29255936 PMC6061467

[fwb13593-bib-0062] Solomon, D. , Lehmann, J. , Kinyangi, J. , Amelung, W. , Lobe, I. , Pell, A. , … Schäfer, T. (2010). Long‐term impacts of anthropogenic perturbations on dynamics and speciation of organic carbon in tropical forest and subtropical grassland ecosystems. Global Change Biology, 13, 511–530. 10.1111/j.1365-2486.2006.01304.x

[fwb13593-bib-0063] Sparling, G. (1992). Ratio of microbial biomass carbon to soil organic carbon as a sensitive indicator of changes in soil organic matter. Soil Research, 30, 195–207. 10.1071/SR9920195

[fwb13593-bib-0064] Stedmon, C. A. , & Markager, S. (2005). Resolving the variability in dissolved organic matter fluorescence in a temperate estuary and its catchment using PARAFAC analysis. Limnology and Oceanography, 50(2), 686–697. 10.4319/lo.2005.50.2.0686

[fwb13593-bib-0065] Steen, A. D. , Quigley, L. N. M. , & Buchan, A. (2016). Evidence for the priming effect in a planktonic estuarine microbial community. Frontiers in Marine Science, 3(6), 10.3389/fmars.2016.00006

[fwb13593-bib-0066] Sun, L. , Perdue, E. M. , Meyer, J. L. , & Weis, J. (1997). Use of elemental composition to predict bioavailability of dissolved organic matter in a Georgia river. Limnology and Oceanography, 42(4), 714–721. 10.4319/lo.1997.42.4.0714

[fwb13593-bib-0067] Tranvik, L. J. (1988). Availability of dissolved organic carbon for planktonic bacteria in oligotrophic lakes of differing humic content. Microbial Ecology, 16, 311–322. 10.1007/BF02011702 24201716

[fwb13593-bib-0068] Vallino, J. J. , Hopkinson, C. S. , & Hobbie, J. E. (1996). Modeling bacterial utilization of dissolved organic matter: Optimization replaces Monod growth kinetics. Limnology and Oceanography, 41, 1591–1609. 10.4319/lo.1996.41.8.1591

[fwb13593-bib-0069] Wang, H. C. , Tian, G. L. , & Chiu, C. Y. (2016). Invasion of moso bamboo into a Japanese cedar plantation affects the chemical composition and humification of soil organic matter. Scientific Reports, 6, 32211 10.1038/srep32211 27558833 PMC4997307

[fwb13593-bib-0070] Warkentin, M. , Freese, H. M. , Karsten, U. , & Schumann, R. (2007). New and fast method to quantify respiration rates of bacterial and plankton communities in freshwater ecosystems by using optical oxygen sensor spots. Applied and Environment Microbiology, 73, 6722–6729. 10.1128/AEM.00405-07 PMC2074954 17766446

[fwb13593-bib-0071] Warton, D. I. , Duursma, R. A. , Falster, D. S. , & Taskinen, S. (2012). smatr 3 – An R package for estimation and inference about allometric lines. Methods in Ecology and Evolution, 3(2), 257–259. 10.1111/j.2041-210X.2011.00153.x

[fwb13593-bib-0072] Weiss, M. , & Simon, M. (1999). Consumption of labile dissolved organic matter by limnetic bacterioplankton: The relative significance of amino acids and carbohydrates. Aquatic Microbial Ecology, 17, 1–12. 10.3354/ame017001

[fwb13593-bib-0073] Wetzel, R. G. (1991). Extracellular enzymatic interactions: storage, redistribution, and interspecific communication In Chróst R. J. (Ed.), Microbial enzymes in aquatic environments (pp. 6–26). New York, NY: Springer‐Verlag.

[fwb13593-bib-0074] Yao, M. , Elling, F. J. , Jones, C. A. , Nomosatryo, S. , Long, C. P. , Crowe, S. A. , … Maresca, J. A. (2016). Heterotrophic bacteria from an extremely phosphate‐poor lake have conditionally reduced phosphorus demand and utilize diverse sources of phosphorus. Environmental Microbiology, 18(2), 656–67. 10.1111/1462-2920.13063 26415900 PMC5872838

[fwb13593-bib-0075] Yeh, T. C. , Liao, C. S. , Chen, T. C. , Shih, Y. T. , Huang, J. C. , Zehetner, F. , & Hein, T. (2018). Differences in N loading affect DOM dynamics during typhoon events in a forested mountainous catchment. Science of the Total Environment, 633, 81–92. 10.1016/j.scitotenv.2018.03.177 PMC6520230 29573694

[fwb13593-bib-0076] Zerulla, K. , Chimileski, S. , Näther, D. , Gophna, U. , Papke, R. T. , & Soppa, J. (2014). DNA as a phosphate storage polymer and the alternative advantages of polyploidy for growth or survival. PLoS One, 9, e94819. 24733558 10.1371/journal.pone.0094819 PMC3986227

