# Supplementary material for: Effects of terrigenous organic substrates and additional phosphorus on bacterioplankton metabolism and exoenzyme stoichiometry
Source: Freshw Biol. 2020 Jul 13;65(11):1973–88. doi: 10.1111/fwb.13593 (PMC7689783; doi:10.1111/fwb.13593)
Supplement: Supplementary file 1 — Supplementary Material [file FWB-65-1973-s001.docx]

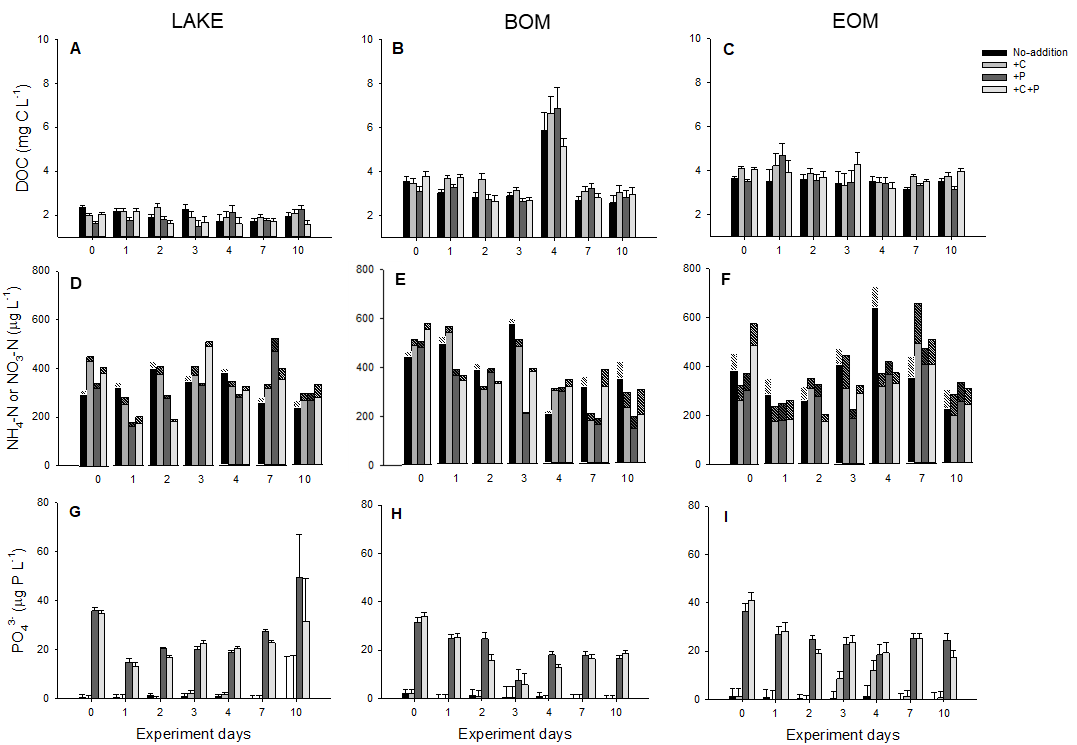
Fig. S1. Time series of dissolved organic carbon (A-C); NO_3_-N and NH_4_-N (D-F, non-shaded and shaded area, respectively); and PO_4_-P (G-I) concentrations of Lake water (left panels), bamboo soil organic matter (middle panels), and evergreen forest soil organic matter (right panels) receiving no nutrient addition, +C, +P, and +C+P addition (bars colours shown in legend). Mean ± 1 S.E.M, n = 3.





Fig. S2. Residuals vs. Fitted value for SMA regressions used in each graph or table.


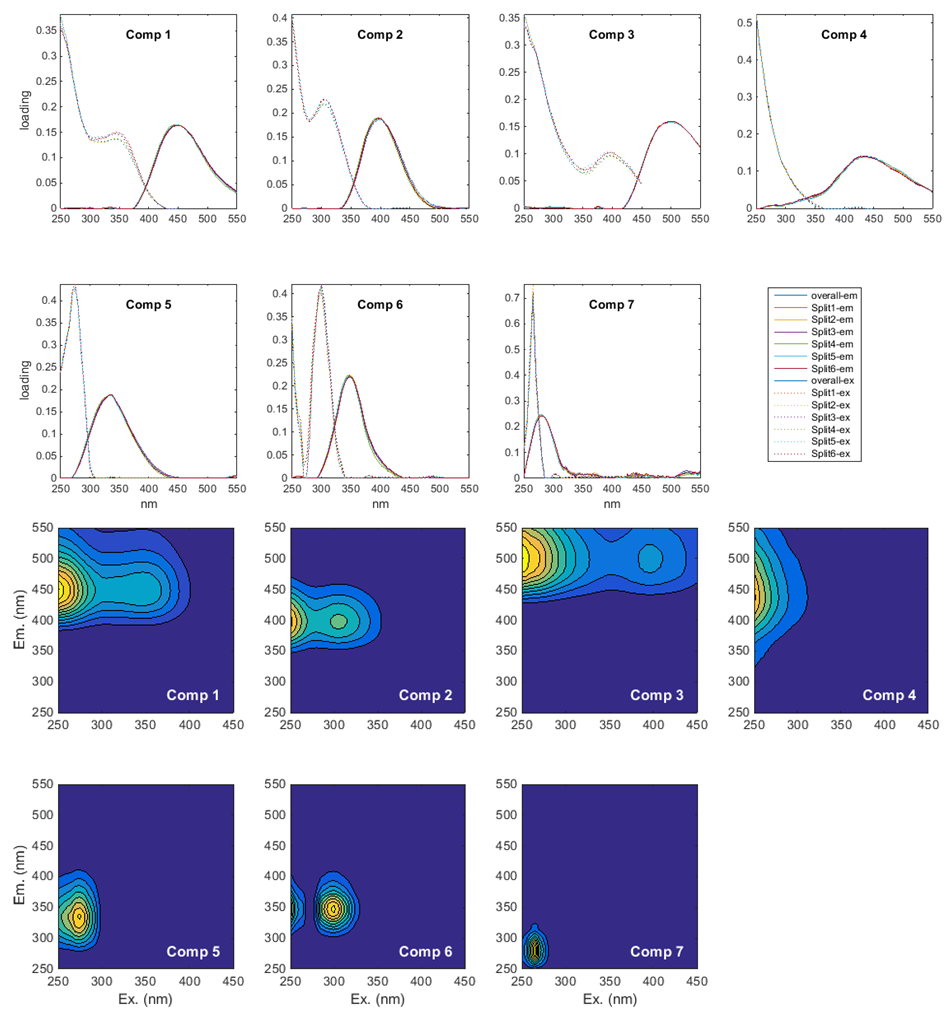
Fig. S3. The validation of the seven components identified from PARAFAC model. Upper two rows presented their spectral properties. Lower two rows presented their EEM fingerprints.


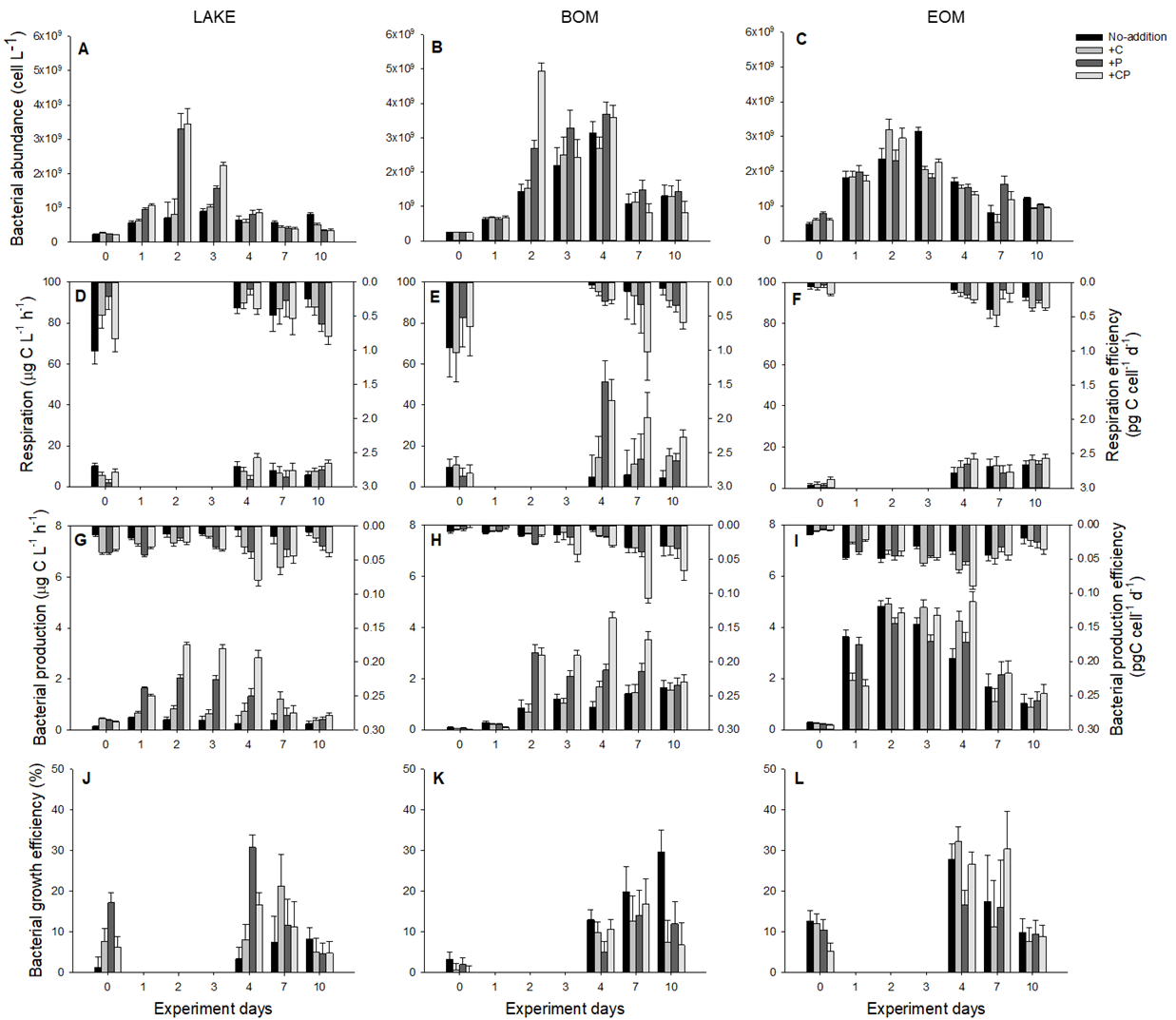
Fig. S4. Time series of bacterial abundance (A-C); respiration efficiency (D-F); and bacterial production (G-I); and bacterial growth efficiency (J-L) of Lake water (left panels), bamboo soil organic matter (middle panels), and evergreen forest soil organic matter (right panels) receiving no nutrient addition, +C, +P, and +C+P addition (bars colours shown in legend). Means ± 1 S.E.M, n = 3.
