# Supplementary material for: Effects of terrigenous organic substrates and additional phosphorus on bacterioplankton metabolism and exoenzyme stoichiometry
Source: Freshw Biol. 2020 Jul 13;65(11):1973–88. doi: 10.1111/fwb.13593 (PMC7689783; doi:10.1111/fwb.13593)
Supplement: Supplementary file 2 — Supplementary Material [file FWB-65-1973-s002.docx]

Table S1. Indicated by peak indices, the optical properties of leachate and the subsequently freeze-dried material extracted from bamboo or evergreen organic soil.

| tOM  Indication | Method | BIX | FI | HIX | T_280_/C ratio |
| --- | --- | --- | --- | --- | --- |
|  |  | Positively related to freshness | Autochthonous or allochthonous source | Positively correlated to the degree of DOM humification | Microbial activity vs. substrate availability |
| BOM | Leachate | 0.547 | 1.118 | 2.969 | 0.544 |
| BOM | Freeze-dried | 0.537 | 1.132 | 2.713 | 0.592 |
| EOM | Leachate | 0.492 | 1.125 | 4.759 | 0.457 |
| EOM | Freeze-dried | 0.471 | 1.069 | 4.517 | 0.415 |

† Em 380 nm/Em_Max_ 420-435 nm, at Ex 310 nm; high BIX (> 1) : autochthonous; low BIX (< 0.6) : allochthonous

Parlanti, E., K. Woerz, L. Geoffroy, and M. Lamotte. 2000. Dissolved organic matter fluorescence spectroscopy

as a tool to estimate biological activity in a coastal zone submitted to anthropogenic inputs. Organic Geochemistry

31:1765-1781.

‡ Em 450 nm/Em 500 nm, at Ex 370 nm; FI < 1.4 : Terrigenous; FI > 1.9 : microbial or autochthonous DOM

McKnight, D. M., Boyer, E. W., Westerhoff, P. K., Doran, P. T., Kulbe, T., Andersen, D. T. 2001. Spectro-

fluorometric characterization of dissolved organic matter for indication of precursor organic material and aroma-

ticity. Limnol Oceanogr 46:38–48.

§ Em_sum_ 435-480 nm/Em_sum_ 300-345 nm and 435-480 nm, at Ex 254 nm

Ohno, T. 2002. Fluorescence Inner-Filtering Correction for Determining the Humification Index of Dissolved

Organic Matter. Environmental Science and Technology 36:742-746.

¶ Peak T_280_ (Ex 270-280 nm/Em 330–368 nm) to Peak C (Ex 330-370 nm/Em 430-460 nm) ratio.

Baker, A. 2001. Fluorescence Excitation-Emission Matrix Characterization of Some Sewage-Impacted Rivers.

Environmental Science and Technology 35:948-953.

Table S2. Physical-chemical and biological parameters of lake water used for the two batch incubation experiments on Day 0 (no treatments added); compared to the Tsengwen reservoir water where the soil used in this experiment was collected.

| Sampling  site | Sampling  date | Water  (°C) | Air  (°C) | Dissolved organic carbon  (mg L^-1^) | Bacterial abundance (cell µg L^-1^) | N-NH4  (µg L^-1^) | N-NO3  (µg L^-1^) | P-PO4  (µg L^-1^) | Bacterial production (ug C L^-1^ h^-1^) | β-glucosidase  (nM h^-1^) | Cellobio-hydrolase  (nM h^-1^) | Alkaline phosphatase  (nM h^-1^) | β-N-acetyl-glucosaminidase  (nM h^-1^) | Leucyl-amino-peptidase  (nM h^-1^) |
| --- | --- | --- | --- | --- | --- | --- | --- | --- | --- | --- | --- | --- | --- | --- |
| Lake Lunz | 26. Oct 2015 | 10.4 | 15 | 2.45 ± 0.53 | 0.45 ± 0.09 | 20.7 ± 14.6 | 264.8 ± 9.7 | 0.67 ± 0.54 | 0.26 ± 0.17 | 90.4 ± 2.12 | 89.5 ± 3.63 | 2713.6 ± 547.49 | 106.3 ± 55.84 | 244.48 ± 185.6 |
|  | 09. Nov 2015 | 9.2 | 15 | 1.88 ± 0.13 | 0.71 ± 0.07 | 25.9 ± 7.52 | 214.3 ± 35.7 | 0.7 ± 0.45 | 0.39 ± 0.33 | 137.6 ± 11.85 | 97.9 ± 35.17 | 2150.9 ± 177.57 | 106.2 ± 10.31 | 172.2 ± 59.97 |
| Tsengwen reservoir | 27. Dec 2014 | 15 | 20 | 3.7 ± 0.12 | N.A. | 39.3 ± 14.7 | 230.5 ± 17.2 | 0.7 ± 0.2 | N.A. | 75.8 ± 0.29 | 79.3 ± 0.55 | 134.9 ± 9.09 | 76.4 ± 0.58 | 43.7 ± 1.77 |

Table S3. The PARAFAC model report.

|  |  |  |  | |  |  | |  |  |  |
| --- | --- | --- | --- | --- | --- | --- | --- | --- | --- | --- |
| Info |  |  |  | |  |  | |  |  |  |
|  | Toolbox | drEEM 0.1.0 | | |  |  | |  |  |  |
|  |  |  |  | |  |  | |  |  |  |
|  |  |  |  | |  |  | |  |  |  |
| Preprocessing | |  |  | |  |  | |  |  |  |
|  | nSample - full dataset | 273 |  | |  |  | |  |  |  |
|  | nSample - modeled dataset | 273 |  | |  |  | |  |  |  |
|  | No. excluded samples | 0 |  | |  |  | |  |  |  |
|  | Excluded samples -indices |  |  | |  |  | |  |  |  |
|  | Scatter Removal | 18 15,15 15,17 18,19 18,1 1 1 1,,3382 | | | | | |  |  |  |
|  | Zapped (Samples,EmRange,ExRange) |  |  | |  |  | |  |  |  |
|  | Fluorescence unit | RU |  | |  |  | |  |  |  |
|  | Scaling | Normalised to unit variance in sample mode | | | | | |  |  |  |
|  |  |  |  | |  |  | |  |  |  |
| PARAFAC model | |  |  | |  |  | |  |  |  |
|  | No. PARAFAC components | 7 |  | |  |  | |  |  |  |
|  | No. Ex wavelengths | 41 |  | |  |  | |  |  |  |
|  | No. Em wavelengths | 151 |  | |  |  | |  |  |  |
|  |  |  |  | |  |  | |  |  |  |
| Validation |  |  |  | |  |  | |  |  |  |
|  | Split_Style | alternating then combine | | | |  | |  |  |  |
|  | Split_NumBeforeCombine | 4 |  | |  |  | |  |  |  |
|  | Split_NumAfterCombine | 6 |  | |  |  | |  |  |  |
|  | Split_Combinations | 1 2 | 3 4 | | 1 3 | 2 4 | | 1 4 | 2 3 |  |
|  | Split_nSample | 137 | 136 | | 137 | 136 | | 137 | 136 |  |
|  | Split_AnalRuns | 5 | 5 | | 5 | 5 | | 5 | 5 |  |
|  | Split_PARAFAC_options | 1E-08 | 2 | | 0 | 0 | | 0 | 0 |  |
|  | Split_PARAFAC_constraints | 2 | 2 | | 2 |  | |  |  |  |
|  | Split_PARAFAC_convgcrit | 1E-08 | 1E-08 | | 1E-08 | 1E-08 | | 1E-08 | 1E-08 |  |
|  | Split_PARAFAC_Initialise | Random |  | |  |  | |  |  |  |
|  | Val_ModelName | Model7 |  | |  |  | |  |  |  |
|  | Val_Source | Model7it_7 | | |  |  | |  |  |  |
|  | Val_Err | 3308.609 |  | |  |  | |  |  |  |
|  | Val_It | 526 |  | |  |  | |  |  |  |
|  | Val_Result | Overall Result= Validated for all comparisons | | | | | |  |  |  |
|  | Val_Splits | AB | CD | | AC | BD | | AD | BC |  |
|  | Val_Comparisons | AB vs CD, | | AC vs BD, | AD vs BC, | |  |  |  |  |
|  | Val_ConvgCrit | 0.000001 |  | |  |  | |  |  |  |
|  | Val_Constraints | nonnegativity | | |  |  | |  |  |  |
|  | Val_Initialise | random |  | |  |  | |  |  |  |
|  | Val_Core | 3.557701 |  | |  |  | |  |  |  |
|  | Val_PercentExpl | 99.87854 |  | |  |  | |  |  |  |
|  | Val_CompSize | 54.63683 | 40.39095 | | 21.9778 | 28.52552 | | 9.529947 | 3.799522 | 0.589731 |
|  | Val_Preprocess | Reversed normalisation to recover true scores | | | | | | |  |  |
